# A Metabolomics-Based Investigation of the Effects of a Short-Term Body Weight Reduction Program in a Cohort of Adolescents with Obesity: A Prospective Interventional Clinical Study

**DOI:** 10.3390/nu15030529

**Published:** 2023-01-19

**Authors:** Antonello E. Rigamonti, Gianfranco Frigerio, Diana Caroli, Alessandra De Col, Silvano G. Cella, Alessandro Sartorio, Silvia Fustinoni

**Affiliations:** 1 Department of Clinical Sciences and Community Health, University of Milan, 20129 Milan, Italy; 2 Department of Clinical Sciences and Community Health, University of Milan, 20122 Milan, Italy; 3 Luxembourg Centre for Systems Biomedicine (LCSB), University of Luxembourg, 6 Avenue Du Swing, L-4367 Belvaux, Luxembourg; 4 Fondazione IRCCS Ca’ Granda Ospedale Maggiore Policlinico, 20122 Milan, Italy; 5 Istituto Auxologico Italiano, Istituto di Ricovero e Cura a Carattere Scientifico (IRCCS), Experimental Laboratory for Auxo-Endocrinological Research, 28824 Piancavallo-Verbania, Italy; 6 Istituto Auxologico Italiano, Istituto di Ricovero e Cura a Carattere Scientifico (IRCCS), Experimental Laboratory for Auxo-Endocrinological Research, 20145 Milan, Italy

**Keywords:** metabolomics, adolescent, obesity, weight reduction programs

## Abstract

Metabolomics applied to assess the response to a body weight reduction program (BWRP) may generate valuable information concerning the biochemical mechanisms/pathways underlying the BWRP-induced cardiometabolic benefits. The aim of the present study was to establish the BWRP-induced changes in the metabolomic profile that characterizes the obese condition. In particular, a validated liquid chromatography–tandem mass spectrometry (LC–MS/MS) targeted metabolomic approach was used to determine a total of 188 endogenous metabolites in the plasma samples of a cohort of 42 adolescents with obesity (female/male = 32/10; age = 15.94 ± 1.33 year; body mass index standard deviation score (BMI SDS) = 2.96 ± 0.46) who underwent a 3-week BWRP, including hypocaloric diet, physical exercise, nutritional education, and psychological support. The BWRP was capable of significantly improving body composition (e.g., BMI SDS, *p* < 0.0001), glucometabolic homeostasis (e.g., glucose, *p* < 0.0001), and cardiovascular function (e.g., diastolic blood pressure, *p* = 0.016). A total of 64 metabolites were significantly reduced after the intervention (at least *p* < 0.05), including 53 glycerophospholipids (23 PCs ae, 21 PCs aa, and 9 lysoPCs), 7 amino acids (tyrosine, phenylalanine, arginine, citrulline, tryptophan, glutamic acid, and leucine), the biogenic amine kynurenine, 2 sphingomyelins, and (free) carnitine (C0). On the contrary, three metabolites were significantly increased after the intervention (at least *p* < 0.05)—in particular, glutamine, trans-4-hydroxyproline, and the octadecenoyl-carnitine (C18:1). In conclusion, when administered to adolescents with obesity, a short-term BWRP is capable of changing the metabolomic profile in the plasma.

## 1. Introduction

The pediatric population is increasingly at risk of becoming severely obese due to unfavorable environmental conditions, including unlimited availability of hypercaloric food and physical inactivity, which are spreading worldwide [1]. In particular, data from the Non-Communicable Diseases Risk Factor Collaboration (NCD-Risk) reveal an increase of ~4–5 times of the prevalence of obesity from 1975 to 2016 when considering children and adolescents living in high-income Western countries [1]. Furthermore, as documented by longitudinal prospective studies that were summarized in a recent review, childhood obesity means, in the future, adulthood obesity, including a series of obesity-related complications, such as type 2 diabetes mellitus (T2DM), cardiovascular diseases, osteoarticular diseases, psychological/psychiatric disorders, and even cancer [2].

There is an urgent need for the early administration of effective anti-obesity interventions such as multidisciplinary body weight reduction programs (BWRPs), consisting of a hypocaloric diet, physical exercise, nutritional education, and psychological support [3,4,5,6]. The success of these interventions (mainly a satisfactory and long-lasting weight loss) depends on a series of factors that are related both to the strict adherence of the patients to a regular clinical follow-up and to the pathophysiological heterogeneity and complexity of the obese state. This view imposes the adoption of a precision therapy (i.e., personalized BWRP) that is capable of overcoming, when present, insulin resistance, chronic inflammation, oxidative stress, mitochondrial dysfunction, hepatic metabolic derangement, gut microbial dysbiosis, epigenetic dysregulation, chronodisruption, and many other unknown molecular mechanisms [7,8].

An investigation of the metabolome, i.e., the omnicomprehensive map of small molecules, commonly known as metabolites, within cells and biofluids (e.g., plasma and urine), which act as substrates/products of enzymatic/non-enzymatic reactions, might be useful to “molecularly” evaluate the effectiveness of any BWRP and, based on positive or negative changes in a cluster of metabolites, to personalize the BWRP administered to the single subject with obesity (e.g., by modifying the composition of the nutrients in the diet) [9,10].

While the metabolomic profile in adults with obesity has been extensively investigated, the number of clinical studies performing metabolomic analyses in children and adolescents with obesity is limited, as evidenced in the systematic review by Handakas et al. [11]. In particular, when considering a pediatric population, despite wide differences in sample processing, metabolome coverage and analytical technique, the most consistent associations between anthropometric measurements (e.g., body mass index (BMI)) and metabolites were observed by considering branched-chain amino acids (BCAAs) (positive association), the aromatic amino acids tyrosine and phenylalanine (positive association), and many other amino acids (both positive and negative) [11]. Other groups of metabolites were measured by clinical studies carried out in children and adolescents with obesity, including acylcarnitines (particularly those of shorter chain length), steroid hormones, glycerophospholipids, sphingolipids, polyamines, peptides, purines, and single metabolites from other classes [11]. Similar to adults with obesity, from these data that were obtained in biologic fluids (plasma, urine and saliva) with the sampled participants at basal conditions, it is possible to envisage a childhood-obesity-specific metabolomic signature [11]. Anyway, if one of the possible clinical applications of metabolomics is the optimization of the BWRP, the research interest should then be the BWRP-induced change of the metabolome.

To the best of our knowledge, few interventional studies have investigated the reversibility of the metabolomic signature that characterizes childhood obesity [12,13,14,15,16,17,18,19]. For instance, while one work did not detect any difference in the serum amino acid profile among children with obesity, measured before and after a 48-week exercise-based program [18], in a 1-year lifestyle intervention administered to a cohort of children with obesity, 17 metabolites were reported to be predictive of weight loss, including arginine, 1 lysophosphatidylcholine (LysPC a C18:0) and 15 long-chain and unsaturated phosphatidylcholines (13 diacyl and 2 acyl-alky PCs) [15]. In a more recent 18-month weight loss intervention in a pediatric population, a cluster of 13 metabolites was identified to change at the end of the study, denoting the involvement of urea and tricarboxylic acid (TCA) cycles and several amino acid metabolic pathways (i.e., arginine, glutamine, glutamate, cysteine, and methionine) [19]. One limitation of these (even) long-lasting studies is the adoption of an out-patient setting, i.e., the enrolment of participants that followed physician-imparted instructions of lifestyle change at home, without the medical supervision that, instead, is ensured in an in-patient setting.

Hence, the aim of the present study was to evaluate the changes in the metabolomic profile in a cohort of adolescents with obesity who underwent a 3-week in-hospital BWRP. A validated targeted metabolomic assay, measuring 188 metabolites belonging to amino acids, biogenic amines, sum of hexoses, acyl-carnitines, glycerophospholipids, and sphingolipids, was used. Our hypothesis is that a short-term BWRP is capable of changing the metabolomic profile in the plasma of adolescents with obesity. Identification of the post-BWRP changed metabolites might allow us to understand the BWRP-induced (mainly) metabolic benefits at the molecular level, i.e., as a consequence of activation/inhibition of specific biochemical pathways.

## 2. Materials and Methods

### 2.1. Study Design

The present clinical study was interventional and prospective, with the administration of a treatment (i.e., the 3-week BWRP) and two follow-up visits (at T0, before starting the BWRP, and T1, at the end of the BWRP). Before enrolment, patient selection was the initial phase of the study to evaluate inclusion/exclusion criteria and to obtain patient’s consent (see below for details). No drop-out was recorded.

### 2.2. Subjects

A cohort of adolescents was selected from the patient population admitted to the Division of Auxology of the Istituto Auxologico Italiano, Piancavallo-Verbania, Italy, for a 3-week in-hospital multidisciplinary BWRP.

The inclusion criteria were subjects with the following characteristics: (1) individuals of both sexes, aged ≤18 years; (2) individuals having a body mass index (BMI) >97th percentile according with age-and sex-specific Italian growth charts [20]; and (3) individuals with or without metabolic syndrome (see below for its definition). The exclusion criteria were (1) secondary causes of obesity (e.g., Prader–Willi syndrome and steroid-induced or medication-induced obesity); (2) individuals with systolic blood pressure (SBP) ≥180 mmHg and diastolic blood pressure (DBP) ≥110 mmHg; (3) cardiovascular disease clinically evident in the previous 6 months; (4) psychiatric, neurological, osteomuscular, or rheumatologic diseases hampering the ability to undertake a (standard) 3-week in-hospital period of metabolic rehabilitation, including physical exercise (see below for details); and (5) individuals (and/or their parents) who refused to sign the consent form.

The study protocol was approved by the Ethical Committee (EC) of the Istituto Auxologico Italiano, IRCCS, Milan, Italy (EC code: 2020_02_18_07; research project code: 01C023); the protocol was explained to the patients and their parents, who gave their written informed consent.

Finally, in this study, we implemented the people-first language, and we strongly encourage other physicians and researchers to do so as a sign of respect and in order to reduce the weight-related bias, following the recommendation of the European Association for the Study of Obesity (EASO) and the Obesity Society (TOS) [21,22].

### 2.3. Body Weight Reduction Program (BWRP)

The BWRP consisted of a 3-week multidisciplinary in-hospital (i.e., full-time staying in the hospital, including the night) metabolic rehabilitation, entailing energy-restricted diet, physical exercise, psychological counseling, and nutritional education. The amount of energy to be given via the diet was calculated by subtracting approximately 500 kcal from the measurement of resting energy expenditure (REE) (see below for details). The diet, in terms of macronutrients, contained approx. 21% proteins, 53% carbohydrates, and 26% lipids; the daily estimated water content was 1000 mL, while the estimated salt content was 1560 mg Na^+^, 3600 mg K^+^, and 900 mg Ca^2+^. Extra water intake of at least 2000 mL/day was encouraged. The diet was served in three meals (breakfast at 07.30 AM, lunch at 12.30 PM, and dinner at 07.30 PM). The breakfast included milk or yogurt with cereals or biscuits; the lunch was composed by a first course of pasta or rice; a second course of beef, chicken, fish, or eggs with a side dish and fruit; and the dinner included a first course of thick soup or pureed vegetables with cereals or rice, a second course of cheese, ham, or fish with a side dish, and fruit.

The physical exercise program consisted of 5 days per week of training, including (i) 1 h dynamic aerobic standing and floor exercise with arms and legs (i.e., squats, step-ups, jump rope, lunges, push-ups, and torso twists), at moderate intensity (monitored through a portable heart rate monitor (Polar RS400SD, Polar Electro Oy, Kempele, Finland)) and under the guidance of a therapist; the intensity of aerobic activities was set at a heart rate corresponding to 60% and 80% of the individual maximal heart rate estimated as 220-age (year); and (ii) either 20–30 min cycloergometer exercise at 60 W (determined through an incremental exercise test performed at the admission to the Hospital), or 3–4 km out-door walking on flat terrain, according to individual capabilities and clinical status.

The subjects also underwent a psychological counselling program (i.e., cognitive behavioral therapy strategies, such as stimulus control procedures, problem-solving and stress management training, development of healthy eating habits, assertiveness and social-skills training, cognitive restructuring of negative maladaptive thoughts, and relapse-prevention training) consisting of two or three sessions per week of individual and/or group psychotherapy performed by clinical psychologists. When possible (1 day per week), additional sessions were also conducted with the parents of the adolescents that were aimed at improving motivation for lifestyle change and interpersonal communication. Furthermore, lectures on the problems and risks of obesity, motivational speech, examples of healthy foods, foods preparation workshops, and group discussions (with or without a supervisor) took place daily.

### 2.4. Resting Energy Expenditure

REE was determined after an overnight fast by means of open-circuit indirect computerized calorimetry (Vmax 29, Sensor Medics, Yorba Linda, CA, USA) with a rigid transparent ventilated canopy.

### 2.5. Anthropometric Measurements

A scale with a stadiometer was used to determine height (with a precision of 0.1 cm) and weight (with a precision of 0.1 kg) (Wunder Sa.Bi., WU150, Trezzo sull’Adda, Italy). Waist circumference (WC) was measured with a flexible tape in standing position, halfway between the inferior margin of the ribs and the superior border of the crista, while the hip circumference (HC) was measured at the largest parts around the buttocks. Body composition was measured by bioimpedance analysis (Human-IM Scan, DS-Medigroup, Milan, Italy) after 20 min of supine resting. BMI (weight in kg divided by height in meters squared), fat mass (FM), and fat-free mass (FFM) were determined in all subjects.

### 2.6. Biological Sample Collection

Blood and 24 h urine samples were collected from patients, following a standardized protocol, at the beginning of the BWRP (T0) and at the end (i.e., 21st day, T1). The same types of tubes and consumables for each cluster of parameters were used throughout the entire duration of the study for improving consistency. 

Blood samples were collected in lithium heparin tubes at around 8:00 AM after an overnight fast. Cells were separated from plasma with centrifugation (20–24 °C for 10 min at 2500 g) within 2 h from the blood collection. Plasma was then transferred in pre-cold tubes and put in ice to preserve the metabolome. Each tube was vortexed for at least 10 s, divided into aliquots, and stored at −20 °C.

Half plasma/urine samples were delivered from the Piancavallo-Verbania to Milan, while keeping the samples frosted, where they were stored at −20 °C until the metabolomics analyses.

### 2.7. Metabolic, Biochemical and Hormonal Evaluation

Total cholesterol (T-C), high-density lipoprotein cholesterol (HDL-C), low-density lipoprotein cholesterol (LDL-C), triglycerides (TG), glucose, insulin, and *C*-reactive protein (CRP) were measured.

Colorimetric enzymatic assays (Roche Diagnostics, Monza, Italy) were used to determine serum T-C, LDL-C, HDL-C, and TG levels. The sensitivities of the assays were 3.86 mg/dL (1 mg/dL = 0.03 mmol/L), 3.87 mg/dL (1 mg/dL = 0.03 mmol/L), 3.09 mg/dL (1 mg/dL = 0.03 mmol/L), and 8.85 mg/dL (1 mg/dL = 0.01 mmol/L), respectively.

The serum glucose level was measured by the glucose oxidase enzymatic method (Roche Diagnostics, Monza, Italy). The sensitivity of the method was 2 mg/dL (1 mg/dL = 0.06 mmol/L). The serum insulin concentration was determined by a chemiluminescent immunometric assay, using a commercial kit (Elecsys Insulin, Roche Diagnostics, Monza, Italy). The sensitivity of the method was 0.2 µU/mL (1 µU/mL = 7.18 pmol/L).

The intra- and inter-assay coefficients of variation (CVs) were as follows: 1.1% and 1.6% for T-C, 1.2% and 2.5% for LDL-C, 1.8% and 2.2% for HDL-C, 1.1% and 2.0% for TG, 1.0% and 1.3% for glucose, and 1.5% and 4.9% for insulin.

CRP was measured by using an immunoturbidimetric assay (CRP RX, Roche Diagnostics GmbH, Mannheim, Germany). The sensitivity of the method was 0.03 mg/dL.

For each patient, the homeostatic model assessment of insulin resistance (HOMA-IR) was calculated according to the following formula: (insulin [μU/mL] × glucose [mmol/L])/22.5 [23].

### 2.8. Evaluation of Blood Pressure

Blood pressure was measured on the right arm, using a sphygmomanometer with appropriate pediatric cuff size, with the subject in a seated position and relaxed condition. The procedure was repeated three times at 10 min intervals in between; the means of the three values for SBP and DBP were recorded.

### 2.9. Definition of Metabolic Syndrome

According to the International Diabetes Federation (IDF) criteria for diagnosis of metabolic syndrome in children and adolescents [24], our patients were considered positive for the presence of metabolic syndrome if they had abdominal obesity (WC ≥ 90th percentile [25] for ages <16 years, and ≥94 cm for males and ≥80 cm for female for ages >16 years) plus two or more of the following factors: (i) increased TG level—≥150 mg/dL (1.7 mmol/L) for ages <16 years and the same cutoff or specific treatment for this lipid abnormality for ages >16 years; (ii) reduced HDL-C—<40 mg/dL (1.03 mmol/L) for males and females for ages <16 years; and <40 mg/dL for males and <50 mg/dL (1.29 mmol/L) for females, or specific treatment for this lipid abnormality for ages >16 years; (iii) increased BP—SBP ≥ 130 mmHg or DBP ≥ 85 mmHg for ages <16 years, and same cutoff or treatment of previously diagnosed hypertension for ages >16 years; and (iv) increased fasting glucose concentration ≥100 mg/dL (5.6 mmol/L) or previously diagnosed type 2 diabetes mellitus for all ages.

### 2.10. Metabolomics Analyses

The metabolomics profile of plasma samples collected from subjects was assessed with a targeted approach, in particular, a liquid chromatography–tandem mass spectrometry method (LC–MS/MS) implementing the AbsoluteIDQ p180 kit (Biocrates Life Sciences AG, Innsbruck, Austria) [26]. With this method, a total of 188 metabolites were quantified, among which were 21 amino acids, 21 biogenic amines, the sum of hexose (H1), 40 acylcarnitine, 15 sphingolipids, and 90 glycerophospholipids (among which were 14 lysophosphatidylcholines (LysoPC), 38 diacylphosphatidylcholine (PC aa), and 38 acyl-alkyl-phosphatidylcholine (PC ae)). The analytical details used in our analyses were extensively reported previously [27]. The details and the list of abbreviations used for the considered metabolites are reported in Supplementary Table S1.

### 2.11. Data Elaboration and Statistical Analyses

Personal characteristics, biochemical parameters, and metabolites’ distribution of the entire cohort at T0 and T1 were reported by using descriptive statistics. Parameters at T0 and T1 were compared by using a paired *t*-test (after natural-log-transformation and standardization by subtracting the mean and dividing by the standard deviation) or chi-squared test for continuous or categorical variables, and a *p*-value < 0.05 was considered statistically significant.

For metabolites, data elaboration was conducted as follows: non-quantifiable metabolites (i.e., lower than the limit of detection, LOD) in more than 50% of the observations were not included in the following statistical elaborations. Then a value equal to the LOD was replaced for all the remaining non-quantifiable measurements. Afterward, metabolite concentrations were log-transformed (base e) and standardized (subtracted by the mean and divided by the standard deviation). For each metabolite, a linear mixed-effects model was built in which the dependent variable was the metabolite concentration and the independent variables with fixed effects were the collection time (before or after the intervention), age, and sex (female or male), while patients were considered as the random intercept variable. The outputs from all the models were collected, and the percentages of variations (∆%) were calculated with the following formula: (exp(β) − 1) × 100, where β was the regression coefficient representing the increase of the metabolite in the difference between a certain category vs. the reference category (in independent categorical variables) or the increase of the metabolite for each unit increase (in independent continuous variables). The *p*-values were adjusted for multiple testing, controlling the false-discovery rate (FDR) according to the method of Benjamini and Hochberg [28], and an FDR *p*-value lower than 0.1 was considered statistically significative. Finally, a volcano plot was created as a visual representation of all the models, plotting the percent of variation (∆%) of each metabolite vs. the negative logarithm of the FDR *p*-value.

Moreover, further similar sets of models were built: in particular, each metabolite was considered as the dependent variable, with age and sex being the independent variables with fixed effects, while patients were considered to be the random intercept variable; finally, other different fixed-effect independent variable were added separately, one at a time, for the set of metabolites, in particular, BMI SDS, WC, HC, waist-to-hip ratio (WHR), FFM, FM, SBP, DBP, heart rate (HR), resting energy expenditure (REE), glycemia, insulin, HOMA-IR, T-C, HDL-C, LDL-C, TG, NEFA, HbA1c, CRP, and presence of metabolomic syndrome (no or yes).

These statistical elaborations were performed by using R (R version 4.1.3, R Foundation, Vienna, Austria) [29] with the Rstudio interface (Version 1.4.1717, RStudio Inc., Boston, MA, USA) and the packages “tidyverse” [30] and “lmerTest” [31].

Furthermore, the entire set of metabolite concentrations (for all the 188 metabolites) was uploaded to MetaboAnalyst [32] to perform Principal Component Analyses (PCA), Partial Least Square Discriminant Analyses (PLSDA), and a pathway analysis. Data were first log-transformed and pareto-scaled. The PLSDA was validated with a cross-validation test (10-fold-CV method) and a permutation test (2000 permutations). The pathway analysis was conducted with the global test enrichment method, the topology analysis out-degree centrality, and the pathway library *Homo sapiens* (KEGG).

## 3. Results

### 3.1. Descriptive Statistics of the Population

The main characteristics of the study population are reported in Table 1. A total of 42 adolescents with obesity (32 females and 10 males) were included, and all of them completed the intervention. The baseline age ranged from 12.5 to 17.9 years, the BMI ranged from 29.60 to 47.12 kg/m^2^, and the BMI standard deviation score (SDS) ranged from 2.09 to 3.79. After the intervention, the BMI ranged from 28.60 to 45.97 kg/m^2^, and the BMI SDS from 1.82 to 3.70.

At the end of the BWRP, when considering the entire population, in addition to weight loss, positive changes in body composition and beneficial metabolic effects and an improvement in the cardiovascular function and the systemic inflammatory state were observed. See Table 1 for details.

### 3.2. Differences in Metabolite Levels Following the BWRP

The complete dataset containing metabolite concentrations and clinical variable measurements is reported in Supplementary Table S2.

**Figure 1:**
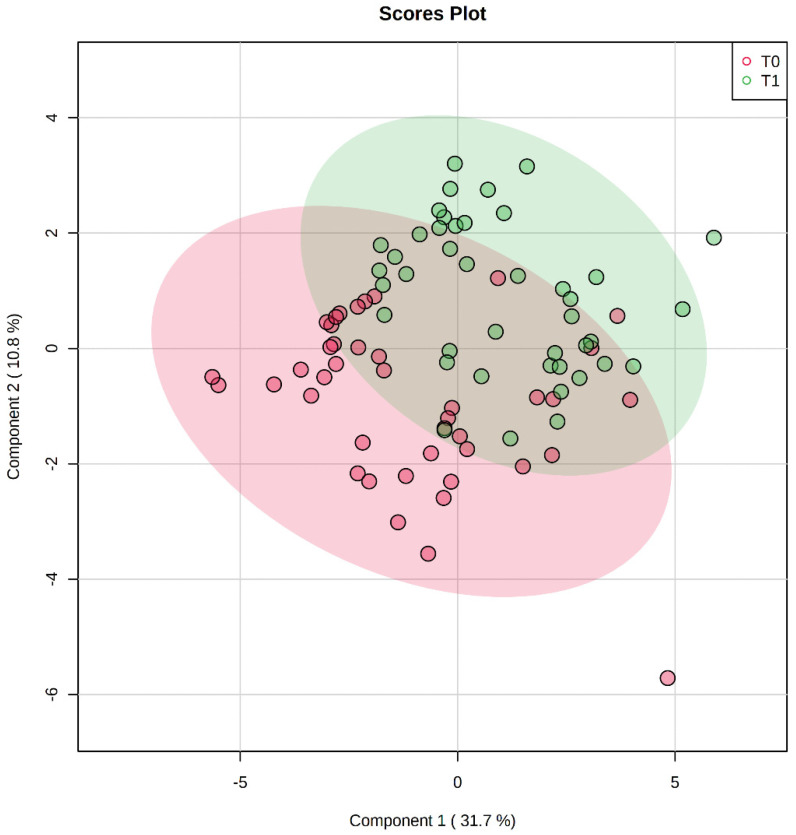
Score plot of the Partial Least Square Discriminant Analyses (PLSDA), considering Component 1 and Component 2. Each patient is represented by two dots: in green at T0 (before BWRP—“pre”) and in red at T1 (after BWRP—“post”). The explained variance for each component is shown in brackets.

The PLSDA is shown in Figure 1, while the PCA is reported in Supplementary Figure S1. The cross-validation and the permutation test of PLSDA are reported in Supplementary Figures S2 and S3, respectively. Overall, the PLSDA model was robust (*p*-value of permutation test equal to 5 × 10^−4^), giving a visual representation of the difference between data obtained at T0 and T1.

**Figure 2:**
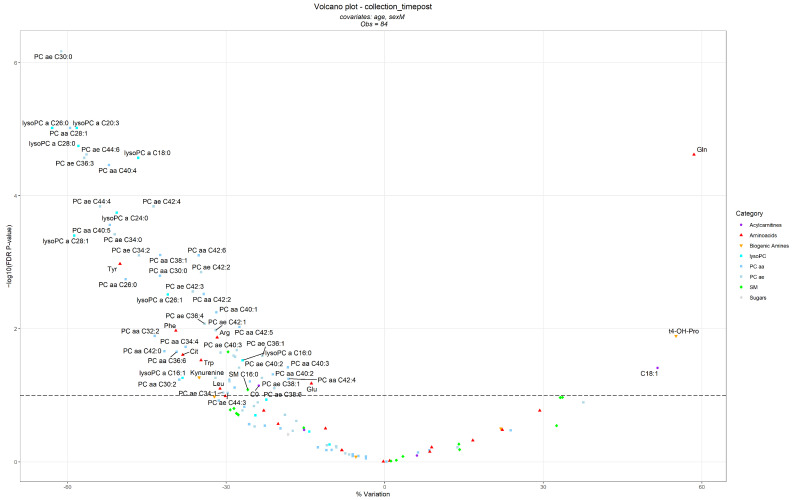
Volcano plot for the linear mixed-effects regression models in which the metabolites (dependent variables) where compared at T1 vs. T0 (independent categorical variable with fixed effects) and corrected for age (independent continuous variable with fixed effects) and sex (independent categorical variable with fixed effects). Patients were considered as the random intercept variable. Each dot represents a metabolite and is displayed based on the % variation at T1 compared toT0 (∆% = (exp(β) − 1) × 100) (x-axis) and the negative logarithm (base 10) of the FDR *p*-value (y-axis). The dashed line represents an FDR *p*-value equal to 0.1.

The results of the linear models assessing the differences in metabolite concentrations before and after the treatment, correcting per age and sex, are summarized in the volcano plot reported in Figure 2, while the complete results are described in Supplementary Table S3.

A total of 64 metabolites were significantly decreased after the intervention, namely 53 glycerophospholipids (23 PCs ae, 21 PCs aa, and 9 lysoPCs); the amino acids tyrosine (tyr), phenylalanine (phe), arginine (arg), citrulline (cit), tryptophan (trp), glutamic acid (glu), and leucine (leu); the biogenic amine kynurenine; 2 sphingomyelins; and acyl-carnitine (C0).

On the contrary, 3 metabolites significantly increased after the intervention, in particular: glutamine (gln), trans-4-hydroxyproline (t4-OH-pro), and the octadecenoyl-carnitine (C18:1).

Table 2 reports descriptive statistics of the significantly different metabolites grouped before (T0) and after (T1) BWRP.

### 3.3. Differences in Metabolite Levels Associated with Other Variables

Supplementary Tables S4–S29 offer an overview of the significant metabolite changes associated with several variables collected in this study, with each model corrected by age and sex, while the detailed results are reported in Supplementary Figures S4–S29.

Considering the FDR *p*-value cutoff of 0.1, no metabolite was significantly associated with the main variables related to adiposity, such as BMI SDS, WHR, FFM, or FM. Some metabolites were associated with HR, REE, insulin, HbA1c, CRP, TG, and T-C. In particular, glutamine was negatively associated with T-C. (See the Supplementary Materials for details.)

### 3.4. Pathway Analysis

**Figure 3:**
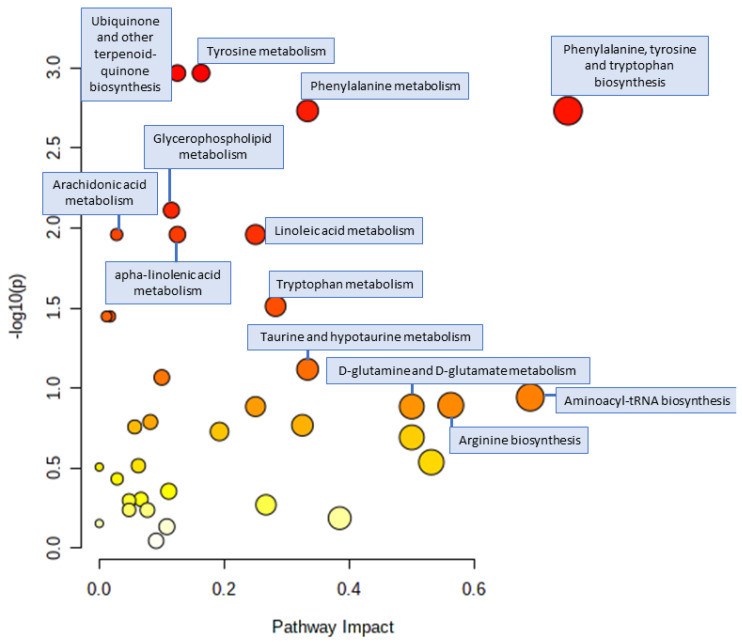
Plot of the pathway analysis. Each dot represents an altered pathway, and they are ordered by pathway impact (x-axis and size) and negative logarithm (base 10) of the *p*-value (y-axis and color). The pathway analysis was performed with a classification between T0 and T1. The pathway analysis was conducted with the global test enrichment method, the topology analysis out-degree centrality, and the pathway library *Homo Sapiens*.

The results of the pathway analysis are reported in Figure 3 and in Supplementary Table S30. The BWRT has the highest impact on the phenylalanine, tyrosine, and tryptophan biosynthesis. Other pathways highly impacted were the aminoacyl-tRNA biosynthesis, the arginine biosynthesis, and the glutamine and glutamate metabolisms, while other pathways with a high significance were the terpenoid-quinone biosynthesis, the glycerophospholipid, the arachidonic acid, the linolenic acid, and the tryptophan metabolisms.

## 4. Discussion

In the present study, a wide targeted metabolomic profiling was carried out in a cohort of adolescents with obesity before and after a 3-week BWRP. The clinical protocol required the hospitalization of the participant, who, for the entire duration of the study, was strictly supervised, particularly for the compliance to the dietetic regimen, with a decrease in confounding environmental factors that are present in clinical studies characterized by a real-world setting.

The main findings consisted of the identification of a cluster of 67 metabolites that were changed at the end of the BWRP. In particular, 64 metabolites were reduced after the intervention, including 53 glycerophospholipids (23 PCs ae, 21 PCs aa, and 9 lysoPCs); the amino acids tyrosine, phenylalanine, arginine, citrulline, tryptophan, glutamic acid, and leucine; the biogenic amine kynurenine; 2 sphingomyelins; and (free) carnitine (C0). On the contrary, 3 metabolites were increased after the intervention, namely glutamine, trans-4-hydroxyproline, and the octadecenoyl-carnitine (C18:1). These changes should be analyzed in the context of the BWRP-induced weight loss and overall cardiometabolic improvement, such as hypoglycemic, hypolipidemic, antihypertensive, and anti-inflammatory effects, which, being well-known, do not deserve further discussion herein. So, in the next paragraphs, we will try to discuss how a “specific” BWRP may reset obesity-disrupted metabolic pathways, forming the basis for a metabolomics-guided BWRP as a precision therapy for the treatment of obesity in adolescents [8].

The main metabolomics changes induced by the short-term BWRP and its possible underlying mechanisms are summarized in Table 3.

In the present study, a plethora of glycerophospholipids was shown to decrease at the end of the BWRP (from −18.2% for PC aa C42:4 to −62.9% for lysoPC a C26:0). The hypocaloric diet and the regular daily practice of physical exercise, essential components of our multidisciplinary BWRP, might have stimulated β-oxidation, with lipolysis and consumption of acyl groups for energy production [33]. Alternatively, as our adolescents were asked, in an in-hospital setting, to follow a low-fat diet, very different from that hypercaloric before the admission to the study, the post-BWRP lipidomic profile might simply represent a missing or reduced intake of fat nutrients [34]. Both mechanisms could also act simultaneously.

LysoPCs are fundamentally derived from PCs during LDL oxidation via either the lecithin–cholesterol acyltransferase (LCAT) or the lipoprotein-associated phospholipase A_2_ (LpPLA_2_) pathway [35]. Since LpPLA_2_ activity has been reported to be increased in children with obesity [35], we can hypothesize that the post-BWRP decrease in plasma lysoPCs may be related to a LpPLA_2_ inhibition, which is congruent with the parallel decrease in plasma LDL. Since lysoPCs exert pro-atherogenic and pro-inflammatory effects and impair insulin signaling [36,37], lipidomic profiling can (at least partially) explain, at the biochemical level, the well-known BWRP-induced cardiovascular benefits [38].

Different from the lipidomic profiling, the BWRP-induced changes (+58.5% for glutamine, and from −13.9% for glutamic acid to −50.1% for tyrosine) in amino acid metabolic pathways are more easily interpretable, since the role of amino acids has been more extensively investigated in obesity (even before the metabolomics advent) [39].

Indeed, over the last decade, BCAA catabolism has increasingly been considered to have an emerging role in the development of insulin resistance in subjects with obesity and T2DM, in whom BCAA levels are considerably elevated in plasma, urine, and tissues [40,41,42,43,44,45]. Although the biochemical mechanisms underlying these findings are not completely known, a dysfunctional BCAA catabolism may be one of the most relevant causative factors [46]. In particular, catabolism of all three BCAA, i.e., leucine, isoleucine, and valine, is located inside the mitochondria, in which two enzymatic reactions occur [47,48]: the first one is the reversible transamination catalyzed by the branched-chain amino acid aminotransferases (BCAT) to form branched-chain α-keto acids (BCKA) [49], while the second one is the irreversible oxidative decarboxylation by the branched-chain α-keto acid dehydrogenase (BCKD) complex, the rate-limiting enzyme of this pathway [50]. Moreover, BCKD is regulated by a process of phosphorylation–dephosphorylation that is carried out by a kinase (BCKDK) inhibiting the enzyme and by a phosphatase (PPM1K) activating the enzyme [51,52]. Interestingly, several studies in animals and humans with obesity point toward the diminishment (or altered function) of the key enzymes involved in BCAA catabolism [50,53,54,55,56]. Thus, increased levels of BCAA in plasma from subjects with obesity are likely to be the result of reduced expression of BCAT [50,57] or decreased BCKD activity via either increased expression of BCKDK [48,50,58,59] or suppression of PPM1K [54,60,61,62]. Furthermore, tissue-specific expressions of BCAA-catabolic enzymes are shown to be dysregulated [53,59,63,64,65,66,67,68,69,70,71,72], especially in adipose tissue [50,73] and the liver [50,65].

In the present study, the plasma levels of leucine were reduced at the end of the BWRP (−31.2%), a finding associated with improvements in body composition (e.g., decreases in BMI and FM) and glucometabolic homeostasis (e.g., decreases in glycemia, insulin, HOMA-IR, and Hb1Ac). Our hypothesis is that BWRP might, at a molecular level, have promoted a reactivation of the BCAA catabolic pathway, particularly in the adipose tissue and skeletal muscle.

Despite that there is evidence that adipose tissue contributes to change circulating BCAA levels, it is suggested to be responsible for less than 5% of whole-body BCAA oxidation [74], meaning that our post-BWRP decrease in plasma BCAA levels should have additional origins [75]. Since gene-expression studies revealed downregulation in multiple steps of the BCAA catabolic pathway in skeletal muscle of subjects with insulin resistance [76,77] and patients with T2DM [78], another explanation of our results might be related to the BWRP-induced increase (though not significant) in FFM (%), with the ensuing stimulation of the mitochondrial bioenergetics, including the BCAA catabolic pathway [79].

The BWRP-related beneficial effects for the mitochondrial function may also explain other metabolomic results of the present study, particularly the post-BWRP decrease in (free) carnitine (−23.8%). Indeed, the end products of BCAA catabolism inside the mitochondria, i.e., succinyl-CoA and acetyl-CoA, are known to enter the TCA cycle, where they act as “anaplerotic” substrates. Defects in BCAA catabolic enzymes may cause the so-called anaplerotic stress, with the ensuing impairment of the mitochondrial respiratory function, resulting in a block of fat oxidation and accumulation of (toxic) catabolic metabolites, including BCAA-derived intermediates and fatty-acid-derived acyl-carnitines [45,76,80,81,82,83,84,85]. Thus, it seems that the BWRP-induced decrease in plasma levels of carnitine in the adolescents included in this study may be a biochemical consequence of a reactivation of the mitochondrial BCAA catabolic pathway and β-oxidation, with an inhibition of the biosynthetic pathway of carnitine, which is not more necessary to transport acyl groups.

Obesity-related accumulation of BCAA in plasma has been reported to interfere with the insulin signaling via activation of the mammalian target of rapamycin (mTOR) pathway, precisely the complex mTOR/p70S6K [86,87,88,89], with leucine as the most potent mTOR activator [90]. In the present study, the BWRP-induced decrease in plasma leucine levels (−31.2%) might be one of the molecular mechanisms underlying the improved glucometabolic homeostasis observed in our adolescents at the end of the intervention. Importantly, despite this consideration, up to date, it is still unknown whether elevated plasma levels of BCAA represent a cause or an effect of insulin resistance [46].

The adipose tissue of obese mice (ob/ob, as well as diet-induced obese) has been demonstrated to produce more glutamate than that of a lean group [91]. The plasma levels of glutamate have been reported to be not only increased, but also associated with visceral adiposity in humans with obesity [92]. This is not surprising when considering the strict linking of glutamate metabolism with BCAA catabolism, with the generation of glutamate and BCKA by BCAT. The obesity-related inhibition, and then the BWRP-induced reactivation of BCAA catabolic pathway, may not only explain the elevated plasma levels of glutamate in obese animal and human models [46], but also the post-BWRP decrease in plasma levels of glutamate in our population of adolescents with obesity. Interestingly, based on the effects of the BWRP on the body composition, a post-BWRP change in plasma glutamate levels seems to depend more on (the increased) skeletal muscle than (the decreased) adipose tissue, further supporting the importance of the BCAA catabolic pathway in the former than the latter tissue (see also above) [76,77,78].

Glutamine is mainly synthesized by glutamine synthetase (GS) and hydrolyzed by glutaminase (GLS); GS catalyzes glutamine biosynthesis by using glutamate and ammonia (NH_3_) as a source, an enzymatic reaction consuming one molecule of ATP [93]. Glutamine, as demonstrated for other amino acids, including BCAA, should be considered not only a simple metabolite, but a metabolic modulator. In this context, plasma levels of glutamine have been reported to be decreased in women with obesity and inversely associated with adiposity [94]. Furthermore, glutamine administration in vitro and in vivo blunts both pro-inflammatory gene and protein levels in adipocytes and adipose tissue specimens, with a reduction in macrophage infiltration in adipose tissue [94]. Metabolomic studies in human adipocytes show that glutamine tones down glycolysis, with a decrease in the synthesis of uridine diphosphate *N*-acetylglucosamine (UDP-GlcNAc), which represents the substrate for the post-translational modification O-linked β-*N*-acetylglucosamine (O-GlcNAc) mediated by the enzyme O-GlcNAc transferase [94,95]. Interestingly, a reduction in glutamine levels corresponds to an interruption of O-GlcNAcylation of nuclear proteins, which results in a decreased pro-inflammatory transcriptional response [94].

Based on the previous considerations, the finding that our BWRP was capable of increasing the plasma levels of glutamine (+58.5%) in a group of adolescents with obesity is extremely relevant because this might explain, at the molecular level, the well-known anti-inflammatory BWRP effectiveness and, presumably, other interventions of metabolic rehabilitation in subjects with obesity [6,96]. The easy measurement of glutamine in plasma might become a novel biomarker to monitor the chronic low-grade inflammatory state in obesity that reportedly plays a critical role in many obesity-related comorbidities [97].

Phenylalanine, an essential amino acid, and tyrosine, a hydroxylation product of phenylalanine metabolism, are largely metabolized in the liver, as the other aromatic amino acid tryptophan (see below). Several animal and human studies have demonstrated that plasma levels of phenylalanine and tyrosine are increased in obesity [98,99,100,101,102,103], with tyrosine being associated with insulin-resistance and T2DM [98,104]. Several biochemical mechanisms have been proposed to explain these findings: (1) the increased plasma levels of the BCAAs compete with those of the aromatic amino acids (such as tyrosine and phenylalanine) for uptake into tissues through a shared transporter, named large neutral amino acid transporter 1 (LAT1) [45,105]; (2) increased liver dysfunction associated with metabolic syndrome (e.g., non-alcoholic fatty liver disease (NAFLD)), which is frequently diagnosed in the pediatric population suffering from obesity, results in decreased catabolism of phenylalanine and tyrosine, leading to their elevated levels in plasma [3,106]; and (3) increased levels of tyrosine are a consequence of the inhibition of tyrosine aminotransferase by cystine, a by-product of the oxidative stress, exacerbated in chronic inflammatory states such as obesity [107,108,109]. Thus, based on the previous considerations, it is not difficult to understand how the BWRP-induced beneficial effects, as demonstrated in the present study and others, including a decrease in BCAAs (with no competition for LAT1) [110], APRI (aspartate transaminase to platelet ratio index, which is used as marker of NAFLD [3], and CRP (*C*-reactive protein, which grossly reveals systemic inflammation) [6,111], whether collectively or solely, can reduce the plasma levels of phenylalanine and tyrosine.

Tryptophan, an essential amino acid, is either used in protein synthesis (anabolism) or metabolized via the kynurenine or methoxyindole pathways. While the kynurenine pathway represents the main route of tryptophan degradation [112] that generates several metabolites collectively called kynurenines, the methoxyindole pathway consists of some enzymatic steps involved in the synthesis of serotonin. The first enzymes of the kynurenine pathway are indoleamine 2,3-dioxygenase 1 (IDO1), indoleamine 2,3-dioxygenase 2 (IDO2), and tryptophan 2,3-dioxygenase (TDO2). Apart from TDO2 and IDO2 expressed in a few tissues, such as liver, kidneys, and lungs, IDO1 is ubiquitous, being expressed in many tissues, including the adipose tissue [113,114]. Interestingly, IDO1 is induced by inflammatory mediators such as interferon-γ (IFN-γ) and lipopolysaccharide (LPS) [112]. Due to the low-grade chronic inflammatory state occurring in obesity, it is not surprising that IDO1 gene expression is increased in the adipose tissue derived from subjects with obesity [115].

In the present study, the BWRP reduced plasma levels of tryptophan (−34.8%) and kynurenine (−35.1%), a biochemical consequence of the BWRP-related anti-inflammatory and anti-adiposity effects, which result in a presumptive downregulation of IDO1 and (at least partial) block of kynurenine pathway, with the ensuing diversion of tryptophan in the methoxyindole pathway and increased synthesis of serotonin [116].

These findings are thought to be relevant in the context of obesity for two reasons: (1) metabolites of kynurenine pathway, particularly xanthurenic acid, have been proposed to be one of the factors predisposing people to insulin-resistance [117] and diabetes [118,119]; (2) the reduced tryptophan availability and serotonin production have been associated with mood disturbances, depression, and impaired satiety, ultimately promoting food intake and obesity [120]. The psychological well-being that is frequently observed in patients with obesity at the end of a BWRP might be explained by the enhancement of serotonin neurotransmission, which, reportedly, is induced by moderate physical exercise, a component of our BWRP [121,122].

In patients with obesity and T2DM, the disrupted protein metabolism may be envisaged in a net catabolic state due to increased loss of nitrogen or negative nitrogen balance, which means amino acid catabolism even without protein restriction. Both epidemiological [123] and experimental [124] studies have reported the existence of an association between high levels of plasma and urinary urea nitrogen and risk of incident T2DM. Biochemically, urea formation mainly occurs in the so-called urea cycle in the liver, involving the substrates arginine, citrulline, and ornithine. The urea cycle consists of several enzymes, which ultimately remove nitrogen from amino acids. Interestingly, some of these enzymes are also involved in the synthesis of nitric oxide, which exerts many physiological functions, predominantly in the cardiovascular system [125]. In this regard, it is known that insulin has impacts on the activity of arginase (key enzyme in urea cycle) and nitric oxide synthase (NOS) [126,127]. Furthermore, amino acids involved in the urea cycle (i.e., arginine, ornithine, and citrulline) have been associated with inflammatory markers and oxidative stress [128,129]. Given the connection between the urea cycle and obesity-related comorbidities such as hyperinsulinemia and T2DM [130], the post-BWRP decrease in the plasma levels of arginine and citrulline that was found in the present study may be interpreted as a reactivation of the urea cycle. Again, the BWRP-induced improvement of glucometabolic homeostasis (e.g., decrease in glycemia and insulinemia) and reduction in the chronic inflammatory state (e.g., decrease in CRP) are likely to play a pivotal role in this process.

Some authors have hypothesized that the disrupted urea cycle in obesity may be related to the incipient NAFLD that is frequently found in subjects suffering from metabolic syndrome [131]. As our BWRP has been demonstrated to improve even only biochemically liver function [3], we cannot rule out that the reactivation of the urea cycle is due to a presumptive BWRP-mediated hepatoprotective effect [132].

Since conflicting results have been reported in the biomedical literature regarding (basal) plasma and urinary levels of arginine, citrulline and ornithine in animal and human models [9], further studies are needed before stating that citrulline is a promising marker to predict metabolic syndrome [133].

In the present study, the plasma levels of hydroxyproline, an amino acid abundantly incorporated in collagen protein [134], increased at the end of the BWRP (+55.1%). A study carried out in a cohort of subjects with obesity showed an elevated urinary excretion of hydroxyproline after starvation, suggesting an active catabolism of collagen proteins present in the connective, rather than bone, tissue [135]. The adolescents included in this study were administered a hypocaloric diet, but they also underwent a program of moderate daily physical exercise, which is reported to be capable of stimulating the remodeling of connective and bone tissues, as documented by the release of a series of biomarkers, such as osteocalcin, PIIINP (Type III Procollagen *N*-terminal Peptide), and hydroxyproline [136,137]. So, based on these considerations, we propose hydroxyproline not as a simple catabolic product, but as a marker of exercise compliance, which might be useful to monitor the effectiveness of any BWRP including physical exercise.

## 5. Strengths and Limitations

Before closing this paper, some strengths and limitations of the present study should be mentioned.

Regarding the strengths, to our knowledge, this is the first study to provide a comprehensive “molecular” discussion of metabolomics changes in response to a short-term BWRP in adolescents with obesity, offering background that can guide clinicians in the implementation of a metabolomics-guided precision medicine in the treatment of pediatric obesity. This can be the first step to optimize and/or personalize, in the clinical practice, the BWRP administered to a subject with obesity, with the possibility of rationally (on a biochemical basis) changing the diet or exercise, in order to obtain more ambitious cardiometabolic benefits.

The robustness of the statistical analysis, together with the control of the main BWRP-related confounding factors (e.g., diet in in-hospital setting; physical exercise in terms of frequency, intensity, time, and type, i.e., the so-called FITT; and psychological support that is often missing or scarcely standardized in some BWRPs), represents another point of strength of the present study. 

The main limitation of the present study is the absence of a control or placebo group. The reasons for this choice rely on the impossibility of enrolling apparently healthy (without obesity) peers to undergo the same experimental protocol as a control group. Moreover, due to ethical considerations, we decided not to carry out a placebo intervention in a group of adolescents with obesity, especially considering that the protocol consisted of in-hospital procedures.

Second, despite our efforts, some methodological aspects still remain that are difficult to standardize (e.g., exercise or psychological support) in qualitative and quantitative terms, but, in the context of the “personalized” BWRP, metabolomics might (or possibly should) suggest how to implement a specific BWRP for the single patient.

Third, future research should investigate larger cohorts and other groups of metabolites. In addition, more complex experimental settings (e.g., after two or three cycles of BWRP per year), comparing BWRPs at different dietary/exercise regimen, or (ambitiously) correlating metabolomic profiles in childhood with cardiometabolic outcomes in adulthood.

## 6. Conclusions

A short-term (3-week) in-hospital multidisciplinary BWRP, determining more than 2% body weight reduction, is capable of improving body composition, glucometabolic homeostasis, and cardiovascular function when administered to adolescents with obesity. At the biochemical level, a specific post-BWRP metabolomic signature can be identified, with a total of 67 metabolites that change in plasma. The interpretation of these changes in terms of metabolic pathways and tissue biochemistry might allow us to understand the BWRP-induced cardiometabolic benefits at the molecular level (e.g., decrease in BMI, FM, HOMA-IR, and CRP). This might represent the basis for implementing a metabolomics-guided BWRP, i.e., a precision therapy for the treatment of obesity [8]. Further studies are mandatory to compare the effectiveness of different BWRPs through a metabolomics-based approach and to identify the metabolites that consistently predict cardiometabolic outcomes even in the long term.

## Figures and Tables

**Table 1:** Descriptive statistics of the study population at T0 and T1 and *p*-value for the comparison between before and after the BWRP.

Parameter	Before BWRP (Mean ± SD)	After BWRP (Mean ± SD)	*p*-Value
N.	42	-	
Sex (F/M)	32/10	-	
Age (years)	15.94 ± 1.33	16.01 ± 1.33	<0.0001
BMI (kg/m^2^)	37.14 ± 4.81	35.60 ± 4.73	<0.0001
BMI SDS	2.96 ± 0.46	2.83 ± 0.52	<0.0001
WHR	0.91 ± 0.08 (missing values: 1)	0.91 ± 0.09 (missing values: 13)	0.025
FFM (kg)	54.85 ± 8.53	53.29 ± 8.05	0.001
FFM %	54.68 ± 5.70	55.41 ± 5.61	0.082
FM (kg)	45.82 ± 9.72	43.28 ± 9.46	<0.0001
FM %	45.33 ± 5.71	44.59 ± 5.61	0.114
SBP (mmHg)	125.95 ± 10.83	124.40 ± 52.15	0.223
DBP (mmHg)	77.50 ± 7.01	74.17 ± 5.94	0.016
HR (bpm)	80.02 ± 12.29	76.74 ± 10.22	0.056
REE (kcal/24 h)	1896.67 ± 447.96	1892.27 ± 444.67 (missing values: 1)	0.948
Glycemia (mg/dL)	87.52 ± 8.71	83.33 ± 6.35	<0.0001
Glycemia (mM)	4.86 ± 0.48	4.62 ± 0.35	<0.0001
Insulin (mU/L)	23.96 ± 13.60 (missing values: 3)	18.06 ± 8.03	0.0001
HOMA-IR	5.31 ± 3.41 (missing values: 3)	3.75 ± 1.86	<0.0001
Total cholesterol (mg/dL)	157.64 ± 28.02	130.00 ± 19.99	<0.0001
HDL-C (mg/dL)	45.05 ± 9.22	37.29 ± 8.19	<0.0001
LDL-C (mg/dL)	100.62 ± 28.51	80.98 ± 20.17	<0.0001
Triglycerides (mg/dL)	110.86 ± 50.14	100.45 ± 34.70	0.092
NEFA (mg/dL)	1.01 ± 0.95 (missing values: 21)	0.72 ± 0.19 (missing values: 22)	0.197
HbA1c (mmol/L)	5.25 ± 0.48	5.08 ± 0.37	<0.0001
CRP (mg/dL)	0.52 ± 0.53	0.33 ± 0.42	<0.0001
Metabolic syndrome	Yes = 11	Yes = 5	0.095
	No = 31	No = 37	

Note: Continuous variables were natural-log-transformed and standardized (by subtracting the mean and dividing by the standard deviation), and then a paired *t*-test was applied, while for the categorical variable “metabolic syndrome”, a chi-squared test was performed. Abbreviations: BMI, body mass index; BMI SDS, body mass index standard deviation score; BWRP, body weight reduction program; CRP, *C*-reactive protein; DBP, diastolic blood pressure; FFM, fat-free mass; FM, fat mass; HbA1c, glycated hemoglobin; HDL-C, high-density lipoprotein cholesterol; HOMA-IR, homeostasis model assessment of insulin resistance; HR, heart rate; LDL-C, low-density lipoprotein cholesterol; NEFA, non-esterified fatty acids; REE, resting energy expenditure; SBP, systolic blood pressure; WHR, waist to hip ratio.

**Table 2:** Descriptive statistics for metabolites at T0 and T1. Only metabolites significantly different at T0 and T1 are reported. Concentrations are shown as median, 5th and 95th percentile, number, and percentage (%) of observations greater than the limit of quantification (LOQ).

Metabolites	Metabolite Category	T0—Before BWRP		T1—After BWRP	
		Median (5th, 95th) (µM)	N > LOQ (%)	Median (5th, 95th) (µM)	N > LOQ (%)
Metabolites significantly increased					
Glutamine (Gln)	Amino Acids	460.0 (147.3, 771.2)	42 (100)	558.0 (245.8, 919.8)	42 (100)
Hydroxyproline (t4-OH-Pro)	Biogenic Amines	14.75 (10.00, 23.51)	35 (83.3)	15.20 (10.61, 29.36)	40 (95.2)
Octadecenoylcarnitine (C18:1)	Acylcarnitines	0.146 (0.076, 0.262)	27 (64.3)	0.181 (0.076, 0.293)	32 (76.2)
Metabolites significantly decreased					
PC ae C30:0	PC ae	0.228 (0.108, 0.339)	39 (92.9)	0.158 (0.108, 0.210)	37 (88.1)
lysoPC a C20:3	lysoPC	1.63 (0.662, 3.26)	41 (97.6)	1.200 (0.302, 1.588)	39 (92.9)
lysoPC a C26:0	lysoPC	0.204 (0.096, 0.316)	34 (81)	0.118 (0.078, 0.204)	35 (83.3)
PC aa C28:1	PC aa	1.94 (1.25, 2.884)	42 (100)	1.455 (1.003, 2.106)	42 (100)
lysoPC a C28:0	lysoPC	0.245 (0.163, 0.483)	32 (76.2)	0.187 (0.082, 0.340)	30 (71.4)
PC ae C44:6	PC ae	0.838 (0.596, 1.289)	42 (100)	0.671 (0.470, 1.058)	42 (100)
lysoPC a C18:0	lysoPC	20.9 (12.42, 44.33)	42 (100)	15.90 (10.22, 36.24)	42 (100)
PC ae C36:3	PC ae	4.315 (2.480, 7.717)	42 (100)	3.275 (2.471, 5.144)	42 (100)
PC aa C40:4	PC aa	4.360 (2.062, 6.650)	42 (100)	3.020 (1.424, 5.292)	42 (100)
PC ae C42:4	PC ae	0.961 (0.374, 2.075)	42 (100)	0.752 (0.325, 1.338)	42 (100)
PC ae C44:4	PC ae	0.278 (0.173, 0.355)	42 (100)	0.220 (0.111, 0.324)	42 (100)
lysoPC a C24:0	lysoPC	0.354 (0.168, 0.512)	30 (71.4)	0.206 (0.168, 0.418)	27 (64.3)
PC aa C40:5	PC aa	5.595 (3.165, 10.570)	42 (100)	4.055 (2.406, 6.841)	42 (100)
PC ae C34:0	PC ae	0.716 (0.405, 1.140)	42 (100)	0.589 (0.334, 0.902)	42 (100)
lysoPC a C28:1	lysoPC	0.225 (0.130, 0.479)	33 (78.6)	0.130 (0.119, 0.351)	29 (69)
PC aa C38:1	PC aa	1.935 (0.309, 4.628)	41 (97.6)	1.250 (0.014, 2.957)	38 (90.5)
PC aa C42:6	PC aa	0.336 (0.236, 0.663)	24 (57.1)	0.236 (0.236, 0.485)	21 (50)
PC ae C34:2	PC ae	6.110 (3.852, 11.530)	42 (100)	5.270 (3.811, 7.327)	42 (100)
Tyrosine (Tyr)	Amino Acids	101.2 (70.61, 145.9)	42 (100)	86.80 (56.09, 127.9)	42 (100)
PC ae C42:2	PC ae	0.607 (0.226, 1.503)	42 (100)	0.496 (0.178, 1.109)	42 (100)
PC aa C30:0	PC aa	1.920 (1.234, 3.438)	42 (100)	1.575 (1.092, 2.932)	42 (100)
PC aa C26:0	PC aa	0.801 (0.522, 0.903)	25 (59.5)	0.732 (0.387, 0.801)	23 (54.8)
PC ae C42:3	PC ae	0.910 (0.327, 1.823)	42 (100)	0.693 (0.256, 1.309)	41 (97.6)
PC aa C42:2	PC aa	0.424 (0.102, 0.988)	41 (97.6)	0.329 (0.086, 0.692)	41 (97.6)
lysoPC a C26:1	lysoPC	0.114 (0.083, 0.229)	24 (57.1)	0.092 (0.080, 0.166)	26 (61.9)
PC aa C40:1	PC aa	0.563 (0.317, 2.020)	27 (64.3)	0.528 (0.382, 1.219)	23 (54.8)
PC ae C36:4	PC ae	11.00 (6.113, 19.60)	42 (100)	9.930 (6.230, 15.19)	42 (100)
PC aa C42:5	PC aa	0.277 (0.033, 0.642)	38 (90.5)	0.227 (0.033, 0.483)	36 (85.7)
PC ae C42:1	PC ae	0.716 (0.172, 1.433)	41 (97.6)	0.462 (0.134, 1.049)	42 (100)
Phenylalanine (Phe)	Amino Acids	98.85 (67.33, 158.1)	42 (100)	89.60 (62.80, 119.0)	42 (100)
PC aa C32:2	PC aa	1.980 (0.650, 3.246)	42 (100)	1.455 (0.848, 2.378)	42 (100)
Arginine (Arg)	Amino Acids	103.0 (65.02, 259.7)	42 (100)	101.5 (46.80, 193.0)	42 (100)
PC aa C34:4	PC aa	0.667 (0.280, 0.999)	42 (100)	0.482 (0.287, 0.834)	42 (100)
PC ae C40:3	PC ae	5.265 (0.534, 12.920)	42 (100)	3.720 (0.429, 9.230)	42 (100)
PC aa C42:0	PC aa	0.649 (0.390, 1.174)	42 (100)	0.570 (0.276, 0.879)	42 (100)
PC aa C36:6	PC aa	0.404 (0.189, 0.643)	42 (100)	0.314 (0.217, 0.490)	42 (100)
SM (OH) C14:1	SM	4.665 (3.021, 6.995)	42 (100)	4.255 (3.070, 6.379)	42 (100)
PC ae C42:5	PC ae	2.780 (1.224, 4.480)	42 (100)	2.380 (1.091, 3.523)	42 (100)
Citrulline (Cit)	Amino Acids	24.25 (13.73, 39.48)	42 (100)	21.05 (12.09, 32.42)	42 (100)
PC ae C38:2	PC ae	5.260 (0.049, 17.32)	38 (90.5)	3.860 (0.049, 10.49)	36 (85.7)
PC ae C36:1	PC ae	10.85 (3.056, 33.05)	42 (100)	8.505 (2.695, 24.28)	42 (100)
PC ae C40:4	PC ae	3.905 (1.260, 8.295)	41 (97.6)	3.245 (0.883, 6.265)	42 (100)
Tryptophan (Trp)	Amino Acids	92.20 (58.41, 121.6)	42 (100)	81.30 (59.16, 108.0)	42 (100)
lysoPC a C16:0	lysoPC	70.75 (45.05, 156.3)	42 (100)	69.90 (38.92, 121.7)	42 (100)
PC aa C40:3	PC aa	0.741 (0.179, 3.099)	42 (100)	0.540 (0.171, 2.579)	42 (100)
PC ae C40:2	PC ae	2.720 (0.686, 7.567)	41 (97.6)	2.075 (0.828, 4.231)	42 (100)
PC aa C40:2	PC aa	1.110 (0.125, 4.271)	42 (100)	0.697 (0.100, 3.349)	42 (100)
((Kynurenine	Biogenic Amines	3.125 (2.414, 4.527)	42 (100)	2.820 (1.980, 4.254)	42 (100)
lysoPC a C16:1	lysoPC	2.38 (1.096, 4.580)	41 (97.6)	2.020 (0.182, 3.628)	37 (88.1)
PC ae C30:2	PC ae	0.135 (0.033, 0.287)	40 (95.2)	0.098 (0.019, 0.194)	38 (90.5)
PC ae C38:1	PC ae	4.720 (0.092, 18.83)	41 (97.6)	3.070 (0.046, 10.39)	37 (88.1)
PC aa C42:4	PC aa	0.301 (0.052, 1.174)	42 (100)	0.276 (0.058, 0.875)	42 (100)
PC aa C30:2	PC aa	0.105 (0.007, 0.222)	39 (92.9)	0.070 (0.007, 0.153)	35 (83.3)
PC aa C42:1	PC aa	0.240 (0.080, 0.512)	41 (97.6)	0.177 (0.090, 0.317)	42 (100)
PC aa C24:0	PC aa	0.268 (0.066, 0.505)	30 (71.4)	0.195 (0.066, 0.392)	31 (73.8)
PC ae C40:1	PC ae	1.545 (0.491, 3.167)	42 (100)	1.195 (0.498, 2.438)	42 (100)
Glutamic acid (Glu)	Amino Acids	154.5 (68.47, 471.1)	42 (100)	139.5 (62.81, 389.4)	42 (100)
L-Carnitine (C0)	Acylcarnitines	35.45 (5, 52.57)	38 (90.5)	33.15 (5.00, 45.17)	36 (85.7)
PC aa C32:1	PC aa	8.125 (4.132, 20.64)	42 (100)	7.065 (4.53, 14.32)	42 (100)
PC ae C38:6	PC ae	3.300 (1.954, 6.958)	42 (100)	3.175 (1.812, 6.681)	42 (100)
Leucine (Leu)	Amino Acids	240.0 (158.4, 339.8)	42 (100)	218.5 (169.2, 304.0)	42 (100)
SM C16:0	SM	88.20 (59.06, 122.6)	42 (100)	82.85 (64.15, 111.9)	42 (100)
PC ae C34:1	PC ae	6.250 (4.400, 9.252)	42 (100)	5.720 (4.386, 7.315)	42 (100)
PC ae C44:3	PC ae	0.134 (0.058, 0.219)	36 (85.7)	0.115 (0.057, 0.155)	32 (76.2)

Abbreviations: PC aa, diacylphosphatidylcholine; PC ae, acyl-alkyl-phosphatidylcholine; SM, sphingolipid.

**Table 3:** Main differences in metabolite concentrations after a short-term BWRP, possible biological underlying mechanisms, and clinical implications in adolescents with obesity.

Difference in Metabolites after the BWRP	Possible Mechanisms or Clinical Implications
↓ glycerophospholipids	↑ β-oxidation ↓ intake of fat nutrients ↓ dyslipidemia
↓ lysoPCs	↓ LpPLA_2_ pathway (related to the decrease of plasma LDL) ↓ atherogenesis
↓ BCAA (in particular leucine)	↑ BCAA catabolic pathway (particularly in the adipose tissue and skeletal muscle) ↑ reversible transamination by BCAT to form BCKA ↑ irreversible oxidative decarboxylation by the BCKD complex (The end products of BCAA catabolism, i.e., succinyl-CoA and acetyl-CoA, enter the TCA cycle as “anaplerotic” substrates) ↓ activation of mTOR with↓ insulin-resistance
↓ tryptophan ↓ kynurenine	↓ IDO1 (ensuing diversion of tryptophan in methoxyindole pathway) ↓ pro-inflammatory transcriptional response ↓ chronic inflammatory state ↑ synthesis of serotonin and ↑ psychological wellbeing
↑ t4-OH-proline	↑ catabolism of collagen proteins (particularly in the connective and bone tissues)
↑ glutamine	↓ synthesis by adipose tissue ↓ *O*-GlcNAcylation of nuclear proteins ↓ pro-inflammatory transcriptional response ↓ chronic inflammatory state
↓ phenylalanine ↓ tyrosine	↑ uptake by LAT1 ↑ activity of tyrosine aminotransferase due to ↓ cystine, a by-product of the oxidative stress ↑ hepatic catabolism (with ↓ NAFLD)
↓ arginine ↓ citrulline	↑ urea cycle due to ↓ fat accumulation in the liver ↑ NOS activity with ↑ endothelial function and cardiovascular benefits

Note: ↓, decreased; ↑, increased. Abbreviations: **BCAA**, branched chain amino acids (leucine, isoleucine, and valine); **BCAT**, branched-chain amino acid aminotransferases; **BCKA**, branched-chain α-keto acids; **BCKD**, branched-chain α-keto acid dehydrogenase; **BWRP**, body weight reduction program; **IDO1**, indoleamine 2,3-dioxygenase 1 (first enzyme of the kynurenine pathway); **LAT1**, large neutral amino acid transporter 1; **LDL**, low-density lipoprotein; **LpPLA_2_**, lipoprotein-associated phospholipase A_2_; **lysoPCs**, lysophosphatidylcholine; **mTOR**, mammalian target of rapamycin; **NAFLD**, non-alcoholic fatty liver disease; **NOS**, nitric oxide synthase; ***O*-GlcNAcylation**, *O*-linked-*N*-acetylglucosaminylation; **t4-OH-proline**, hydroxyproline; **TCA**, tricarboxylic acid cycle.

## Data Availability

The datasets used and/or analyzed in the present study are available in the Supplementary Materials.

## Supplementary Materials

The following supporting information can be downloaded at https://www.mdpi.com/article/10.3390/nu15030529/s1. Table S1: Complete information about all the 188 considered metabolites, including the abbreviations used, the HMDB codes, and the CAS registry numbers, when available; Table S2: Database with all the data considered for this work, including both the subject’s anthropometric and clinical characteristics, and metabolite concentrations; Tables S3–S29: Complete results of the linear models with mixed effects. For all the models, the dependent variables were the log-transformed and standardized concentrations of the metabolites, the independent variables with fixed effects were age and sex (female as reference), while patients were considered as the random intercept variable. Within the models reported in each table, an additional independent variable with fixed effect was added, in particular: collection time (before or after the intervention) (Table S3), BMI SDS (Table S4), waist circumference (Table S5), hips circumference (Table S6), waist-to-hip ratio (Table S7), fat-free mass (Table S8), fat mass (Table S9), systolic blood pressure (Table S10), diastolic blood pressure (Table S11), heart rate (Table S12), resting energy expenditure (Table S13), glycemia (Table S14), insulin (Table S15), homeostasis model assessment of insulin resistance (Table S16), total cholesterol (Table S17), high-density lipoprotein (Table S18), low-density lipoprotein (Table S19), triglycerides (Table S20), non-esterified fatty acids (Table S21), glycated hemoglobin (Table S22), *C*-reactive protein (Table S23), cortisol measured at about 8.00 (Table S24), cortisol measured at about 15.00 (Table S25), adrenocorticotropic hormone measured at about 8.00 (Table S26), adrenocorticotropic hormone measured at about 15.00 (Table S27), cortisol measured in 24 h urine samples (Table S28), presence of metabolic syndrome (NO as reference) (Table S29). The columns of the table report: the dependent variables (Dependent), the independent variables with fixed effects (Independent), the total number of observations, i.e.: the number of plasma samples analysed (N_observations), the number of groups of observations, i.e.: the number of subjects (N_groups), the regression coefficient (beta), the 95% coefficient interval of the beta (beta_confint2.5 and beta_confint97.5), the standard error of the beta (SE), the adjusted marginal and conditional R2 (adj_R_sqrd_marginal and adj_R_sqrd_conditional), the *p*-value of the beta (*p* value), *p*-value of the beta corrected for the false discovery rate (FDR_*p* value), the negative logarithm (base 10) of the FDR *p*-value (negative_log10fdr), the variation percentage calculated with the formula (exp(β) − 1) × 100) (variation_perc), and the category of the considered metabolites (Category); Table S30: Complete results of the pathway analysis performed considering metabolite levels and the classification between T0 and T1, conducted with the global test enrichment method, the topology analysis out-degree centrality, and the pathway library Homo sapiens (KEGG). Results include total number of compounds in the pathway (Total Cmpd), number of our considered metabolites for that pathway (Hits), *p*-value (Raw p), the negative logarithm (base 10) of the *p*-value (-LOG10(@p)), Holm–Bonferroni adjusted *p*-value (Holm adjust), the false discovery rate *p*-value (FDR), and the impact (impact); Figure S1: Visual representation of the plasma metabolite concentrations through principal component analysis (PCA), showing the relationship between principal component 1 (x-axis) and principal component 2 (y-axis). Each subject is represented by two dots: green for the blood sample analysed before the BWRP (T0, pre) and in red for the sample analysed after the BWRP (T1, post); Figure S2: Cross-validation of the PLSDA model showed in Figure 1; Figure S3: Permutation test statistics (2000 permutation) of the PLSDA model showed in Figure 1; Figures S4–S29: Volcano plots for the linear mixed-effects regression models in which the metabolites (dependent variables) where compared with the different independent variables considered (complete results from the models are reported in Tables S4–S29), and are corrected for age (independent continuous variable with fixed effects) and sex (independent categorical variable with fixed effects), while patients were considered as the random intercept variable. The independent variable considered in each figure is: BMI SDS (Figure S4), waist circumference (Figure S5), hips circumference (Figure S6), waist-to-hip ratio (Figure S7), fat-free mass (Figure S8), fat mass (Figure S9), systolic blood pressure (Figure S10), diastolic blood pressure (Figure S11), heart rate (Figure S12), resting energy expenditure (Figure S13), glycemia (Figure S14), insulin (Figure S15), homeostasis model assessment of insulin resistance (Figure S16), total cholesterol (Figure S17), high-density lipoprotein (Figure S18), low-density lipoprotein (Figure S19), triglycerides (Figure S20), non-esterified fatty acids (Figure S21), glycated hemoglobin (Figure S22), *C*-reactive protein (Figure S23), cortisol measured at about 8.00 (Figure S24), cortisol measured at about 15.00 (Figure S25), adrenocorticotropic hormone measured at about 8.00 (Figure S26), adrenocorticotropic hormone measured at about 15.00 (Figure S27), cortisol measured in 24 h urine samples (Figure S28), presence of metabolic syndrome (no as reference) (Figure S29). Each dot represents a metabolite and is displayed based on the % variation at T1 compared to T0 (∆% = (exp(β) − 1) × 100) (x-axis) and the negative logarithm (base 10) of the FDR *p*-value (y-axis). The dashed line represents an FDR *p*-value equal to 0.1.

## Abbreviations

AA, amino acids; AC, acyl-carnitine; APRI, aspartate transaminase to platelet ratio index; Arg, arginine; ATP, adenosine triphosphate; BA, biogenic amines; BCAA, branched-chain amino acids; BCAT, branched-chain amino acid aminotransferases; BCKA, branched-chain α-keto acids; BCKD, branched-chain α-keto acid dehydrogenase; BCKDK, Branched Chain Keto Acid Dehydrogenase Kinase; BMI, body mass index; BMI SDS, body mass index standard deviation score; BWRP, body weight reduction program; C0, L-carnitine; C18:1, octadecenoylcarnitine; EC, Ethical Committee; Cit, citrulline; CRP, *C*-reactive protein; DBP, diastolic blood pressure; FFM, fat-free mass; FM, fat mass; Gln, glutamine; GLS, glutaminase; Glu, Glutamic acid; GS, glutamine synthetase; HbA1c, glycated hemoglobin; HC, hip circumference; HDL, high density lipoprotein; HOMA-IR, homeostasis model assessment of insulin resistance; HR, heart rate; IDO1, indoleamine 2,3-dioxygenase 1; IDO2, indoleamine 2,3-dioxygenase 2; IFN-γ, interferon-γ; LAT1, large neutral amino acid transporter 1; LC–MS/MS, liquid chromatography–tandem mass spectrometry; LDL, low density lipoprotein; Leu, leucine; LpPLA2, lipoprotein-associated phospholipase A_2_; LPS, lipopolysaccharide; lysoPC, lysophosphatidylcholine; mTOR, mammalian target of rapamycin; NAFLD, non-alcoholic fatty liver disease; NCD-Risk, Non-Communicable Diseases Risk Factor Collaboration; NEFA, non-esterified fatty acids; NOS, nitric oxide synthase; O-GlcNAc, O-linked β-*N*-acetylglucosamine; PC aa, diacylphosphatidylcholine; PC ae, acyl-alkyl-phosphatidylcholine; Phe, phenylalanine; PIIINP, Type III Procollagen *N*-terminal Peptide; PPM1K, Protein phosphatase 1K; REE, resting energy expenditure; SBP, systolic blood pressure; SM, sphingolipid; T2DM, type 2 diabetes mellitus; t4-OH-pro, hydroxyproline; T-C, total cholesterol; TCA, tricarboxylic acids; TG, triglycerides; TDO2, tryptophan 2,3-dioxygenase; Trp, tryptophan; Tyr, tyrosine; UDP-GlcNAc, uridine diphosphate *N*-acetylglucosamine; WC, waist circumference; WHR, waist to hip ratio.
