# Supplementary figures and images for: A Metabolomics-Based Investigation of the Effects of a Short-Term Body Weight Reduction Program in a Cohort of Adolescents with Obesity: A Prospective Interventional Clinical Study
Source: Nutrients. 2023 Jan 19;15(3):529. doi: 10.3390/nu15030529 (PMC9921209; doi:10.3390/nu15030529)

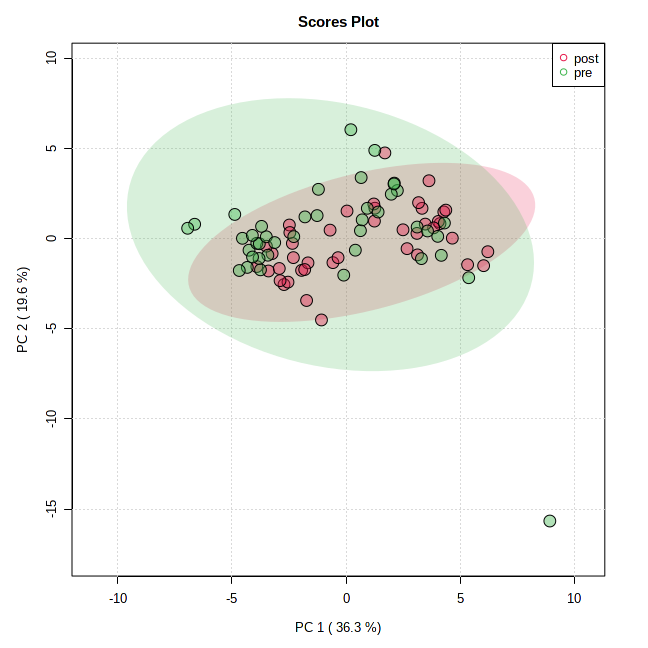

Supplement: Supplementary file 1 [file nutrients-15-00529-s001.zip › 2022-07-12_PRG_Supplementary_material_GF/S01_PCA.png]

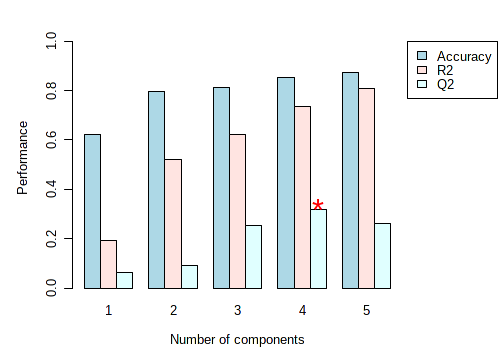

Supplement: Supplementary file 1 [file nutrients-15-00529-s001.zip › 2022-07-12_PRG_Supplementary_material_GF/S02_pls-cross_validation.png]

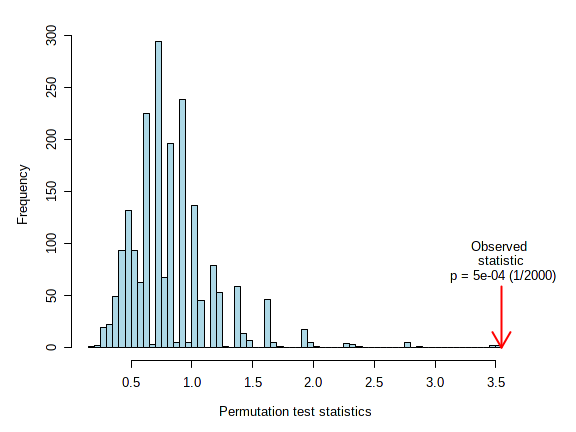

Supplement: Supplementary file 1 [file nutrients-15-00529-s001.zip › 2022-07-12_PRG_Supplementary_material_GF/S03_permutation_statistic.png]

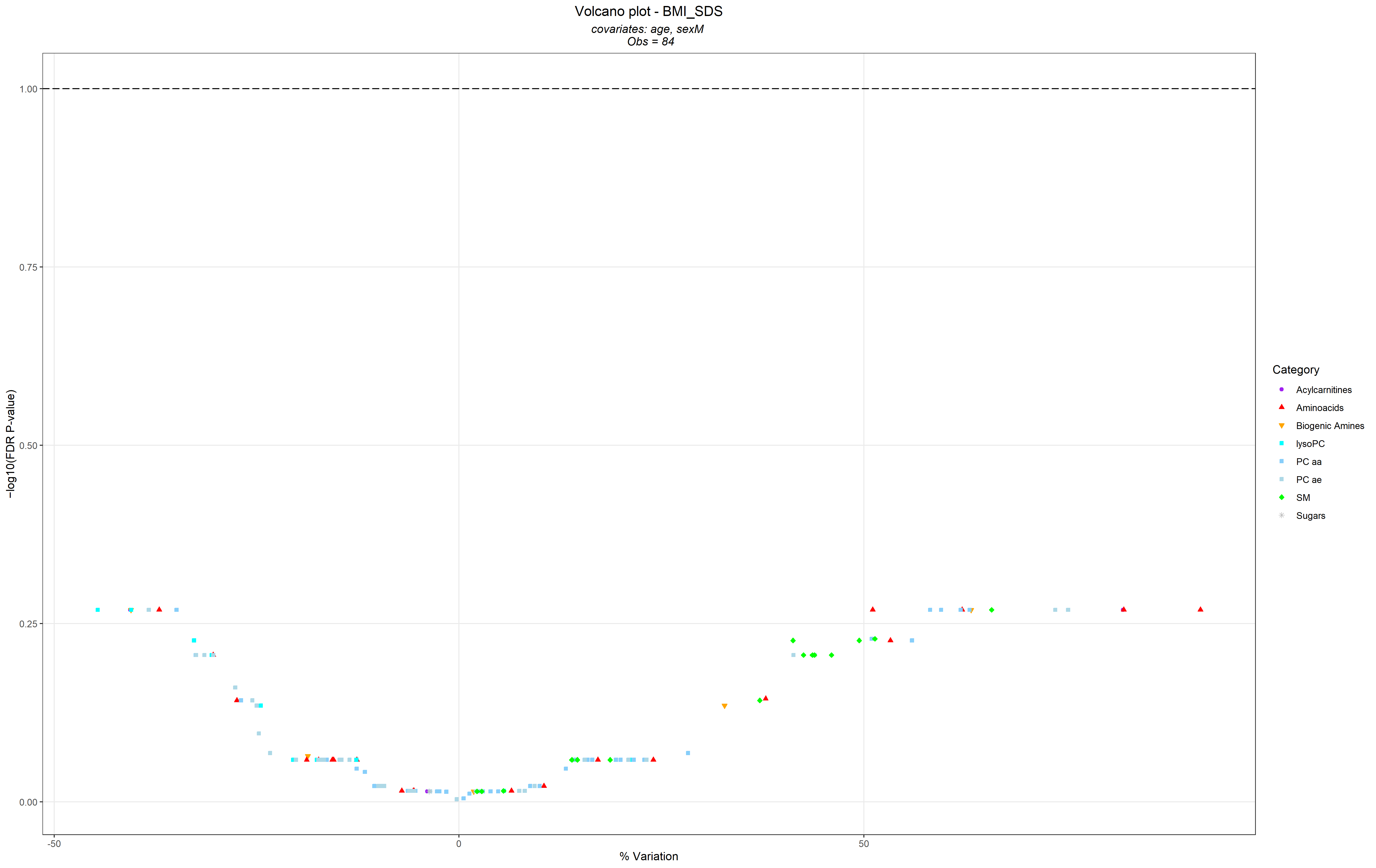

Supplement: Supplementary file 1 [file nutrients-15-00529-s001.zip › 2022-07-12_PRG_Supplementary_material_GF/S04_PRG_lmer_BMI_SDS.png]

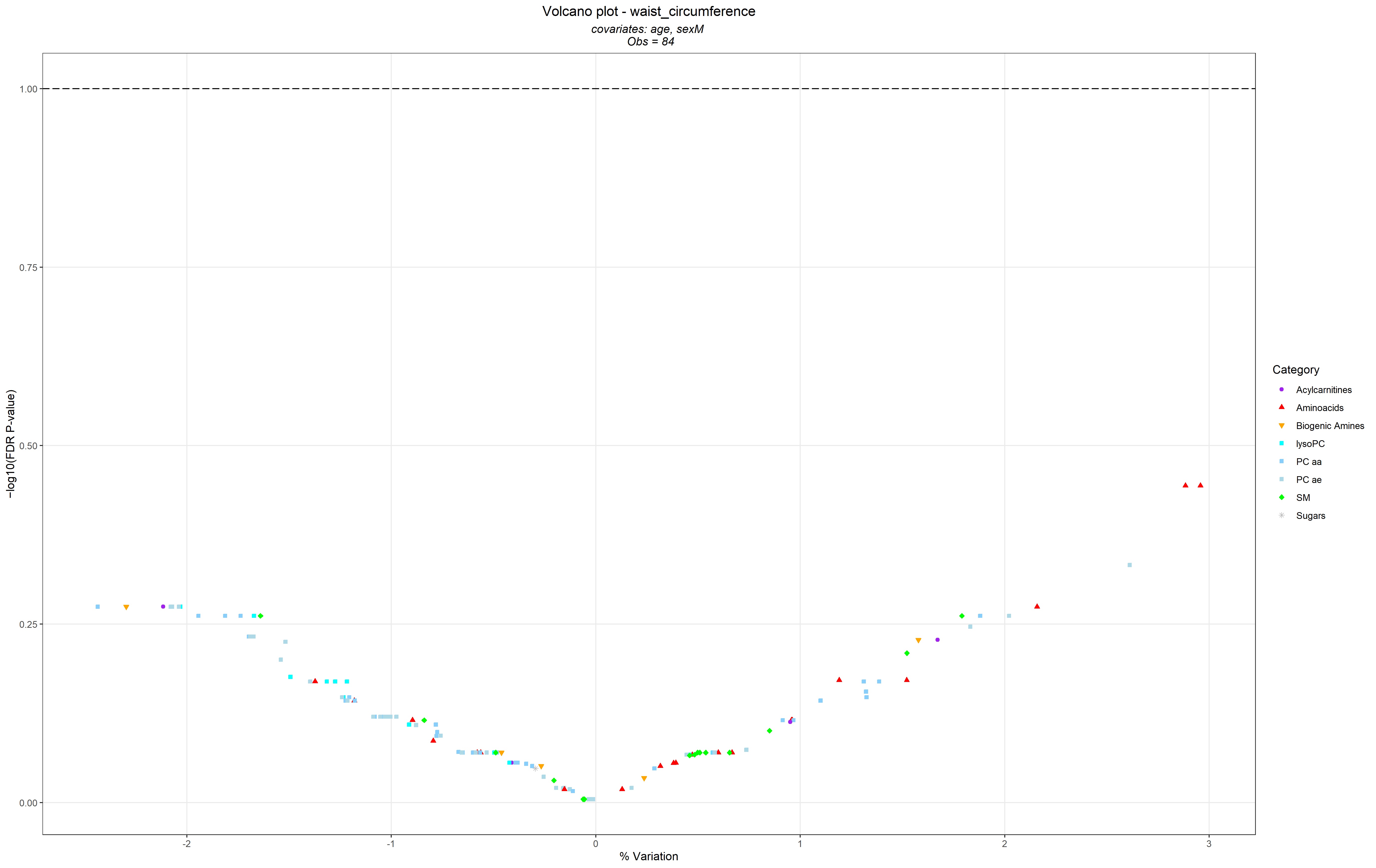

Supplement: Supplementary file 1 [file nutrients-15-00529-s001.zip › 2022-07-12_PRG_Supplementary_material_GF/S04_PRG_lmer_waist_circumference.png]

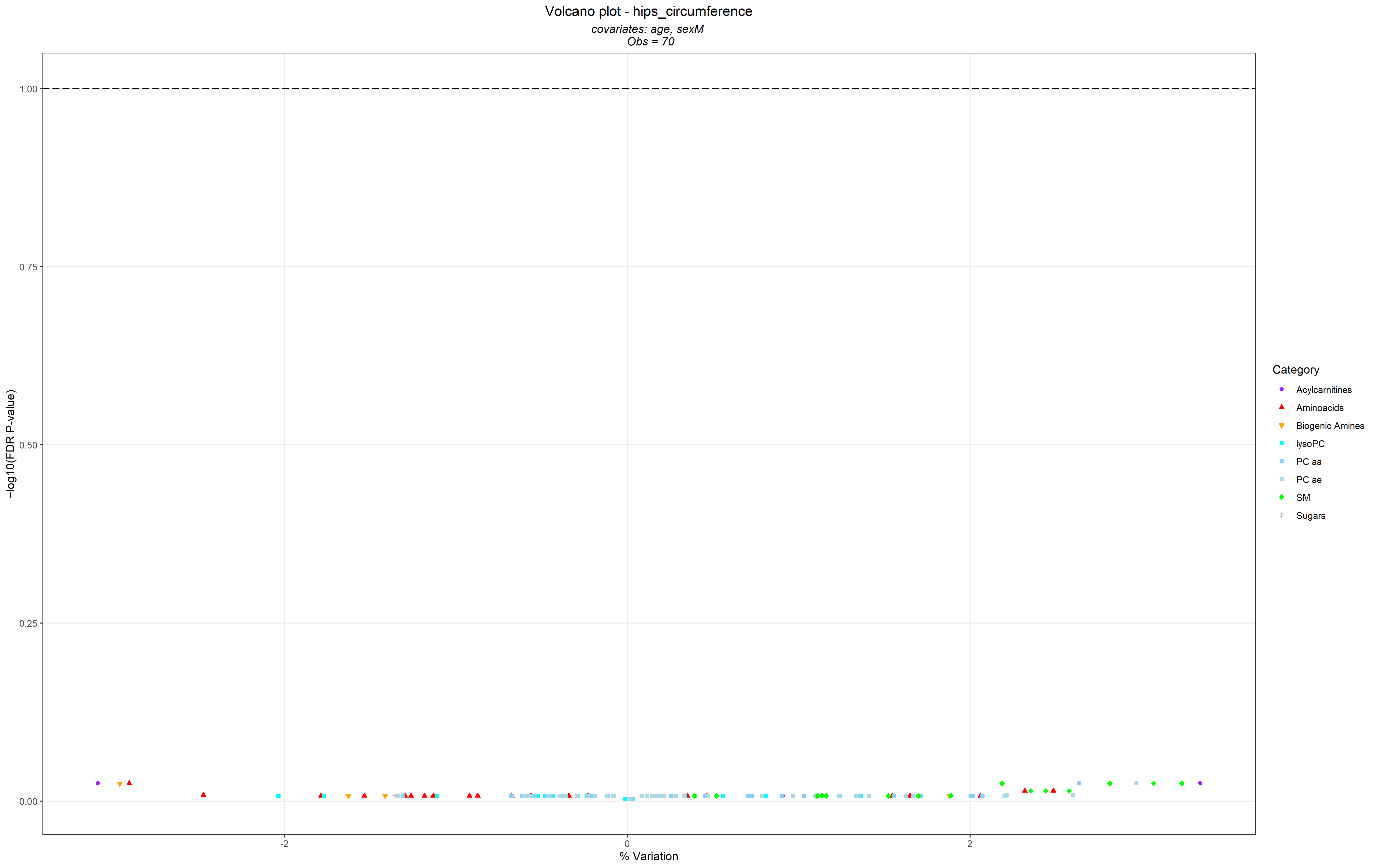

Supplement: Supplementary file 1 [file nutrients-15-00529-s001.zip › 2022-07-12_PRG_Supplementary_material_GF/S06_PRG_lmer_hips_circumference.png]

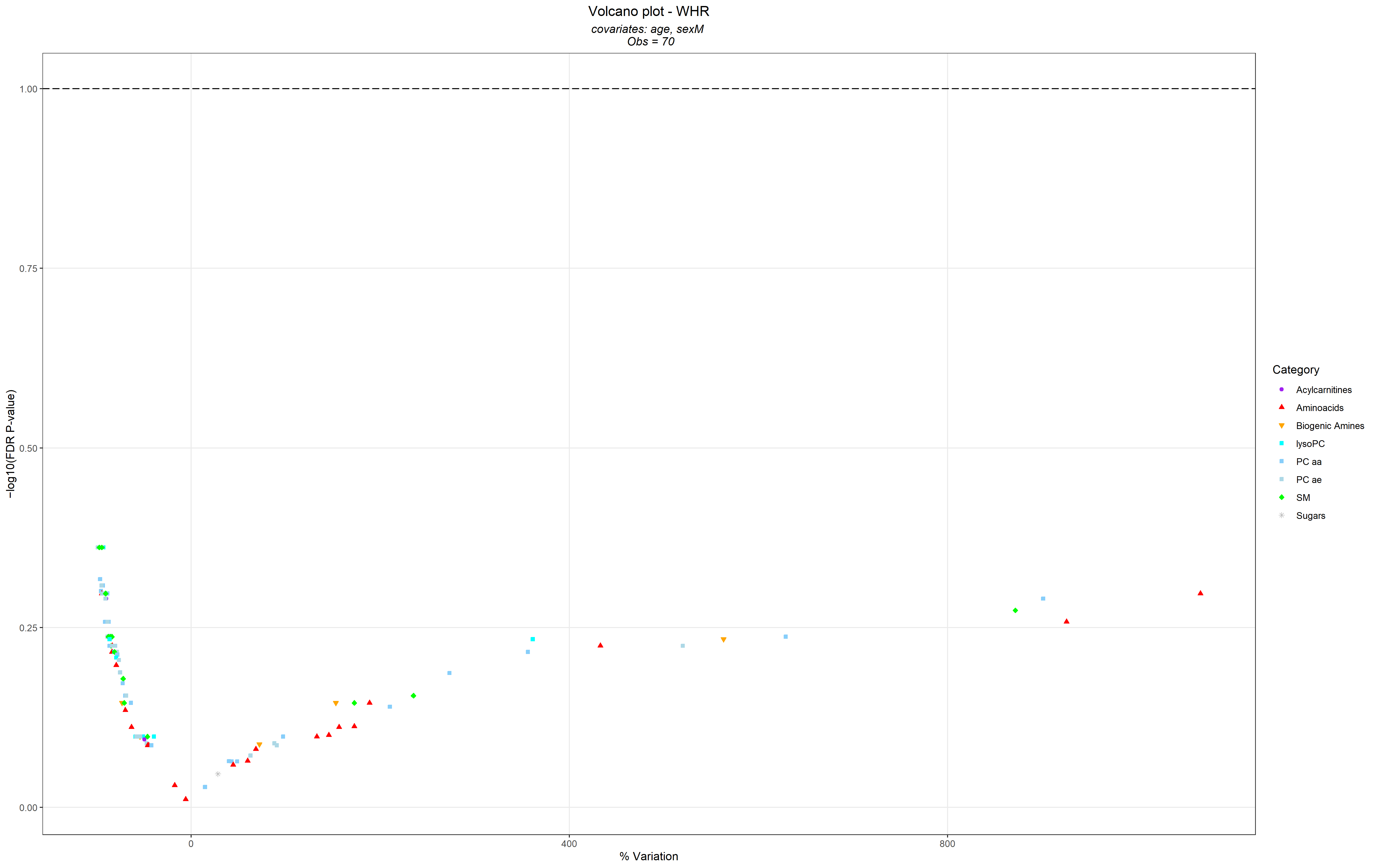

Supplement: Supplementary file 1 [file nutrients-15-00529-s001.zip › 2022-07-12_PRG_Supplementary_material_GF/S07_PRG_lmer_WHR.png]

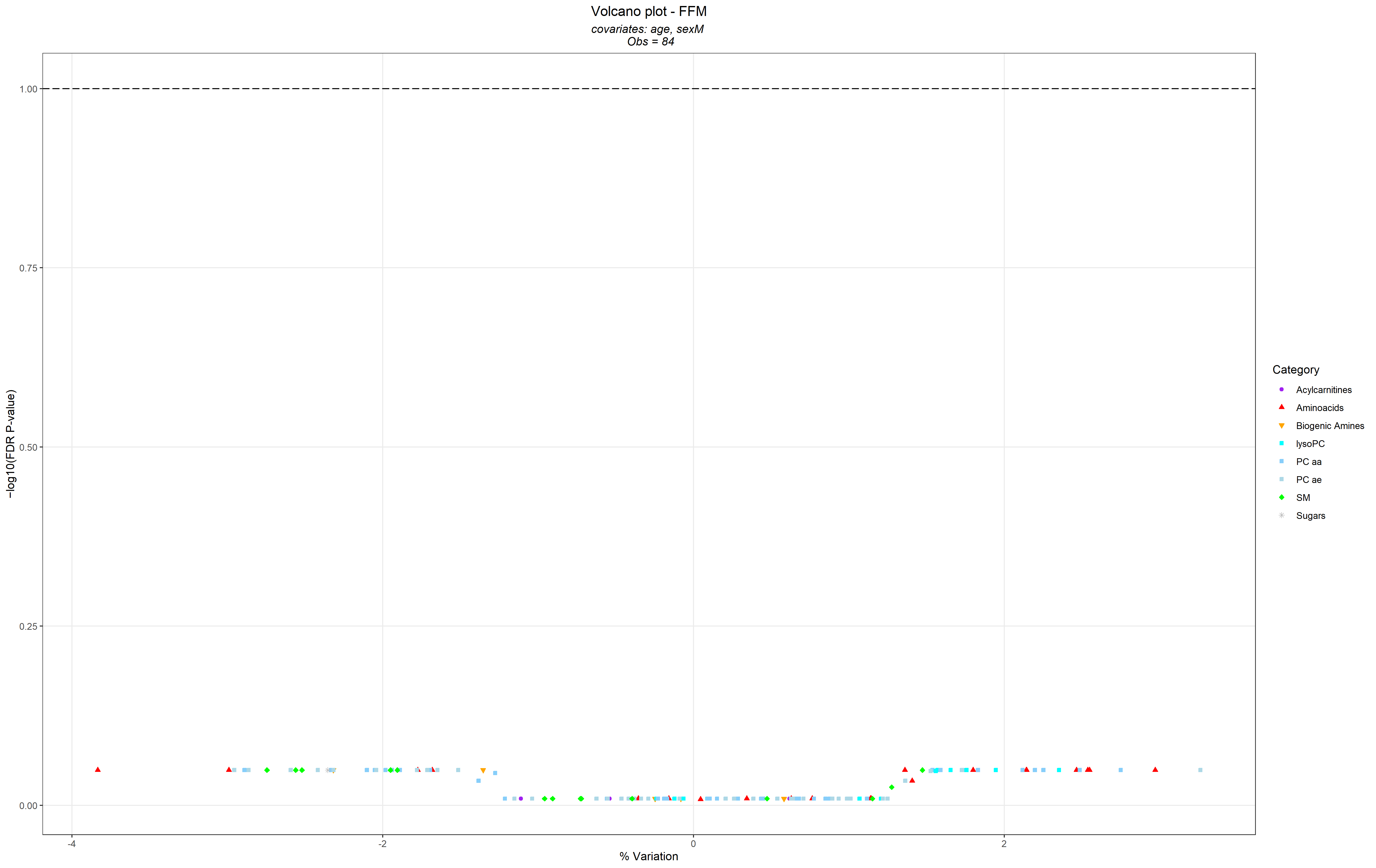

Supplement: Supplementary file 1 [file nutrients-15-00529-s001.zip › 2022-07-12_PRG_Supplementary_material_GF/S08_PRG_lmer_FFM.png]

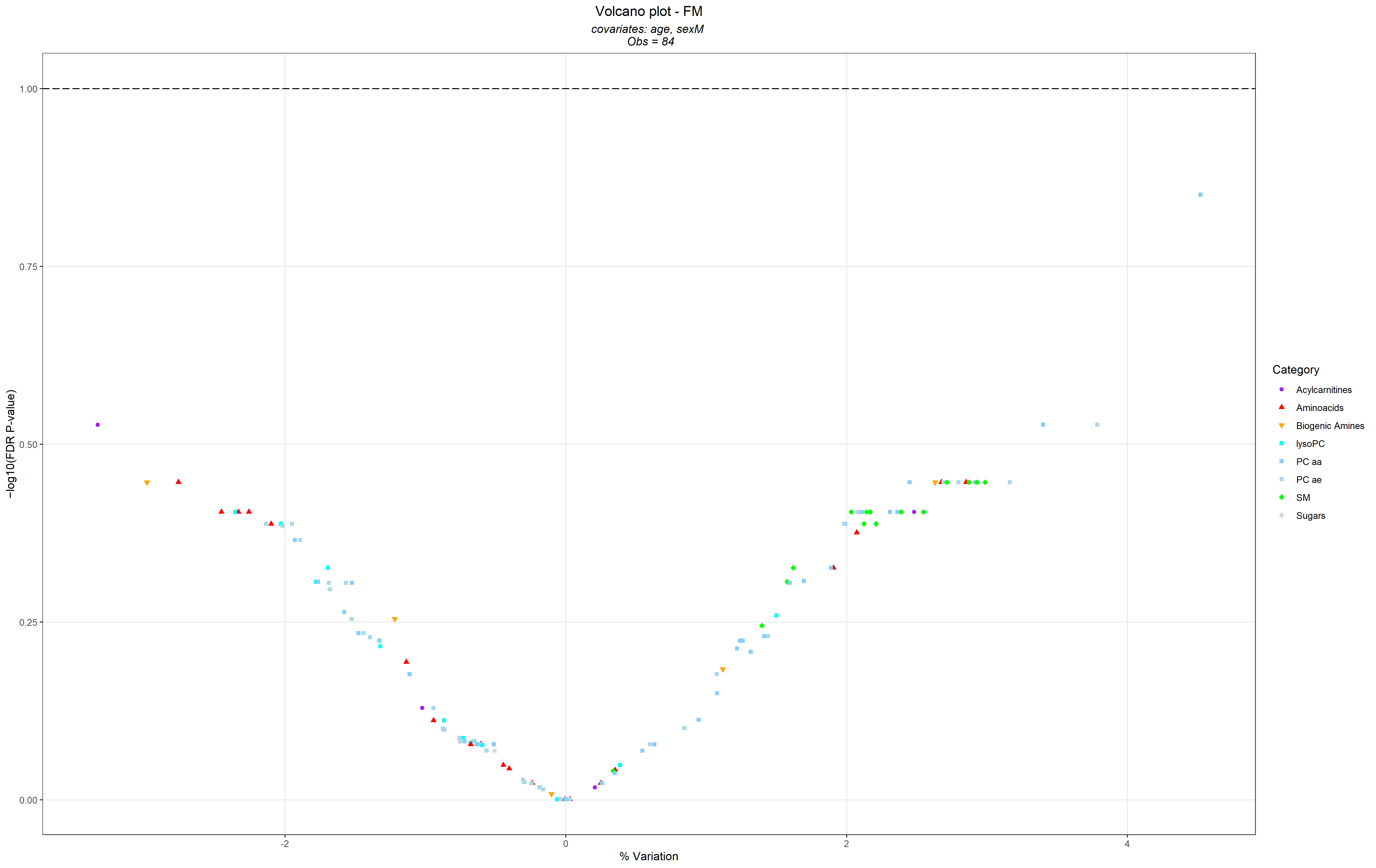

Supplement: Supplementary file 1 [file nutrients-15-00529-s001.zip › 2022-07-12_PRG_Supplementary_material_GF/S09_PRG_lmer_FM.png]

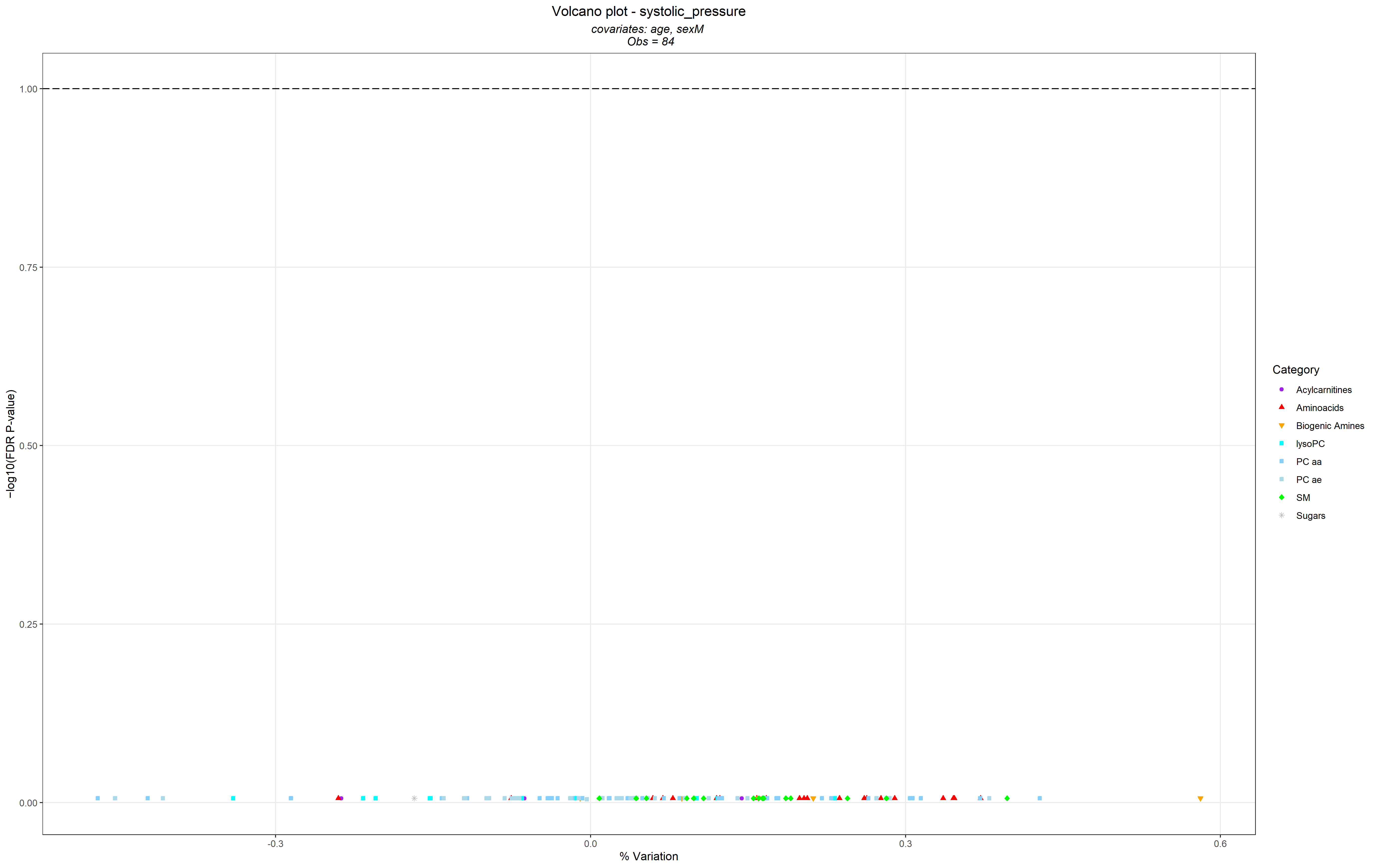

Supplement: Supplementary file 1 [file nutrients-15-00529-s001.zip › 2022-07-12_PRG_Supplementary_material_GF/S10_PRG_lmer_systolic_pressure.png]

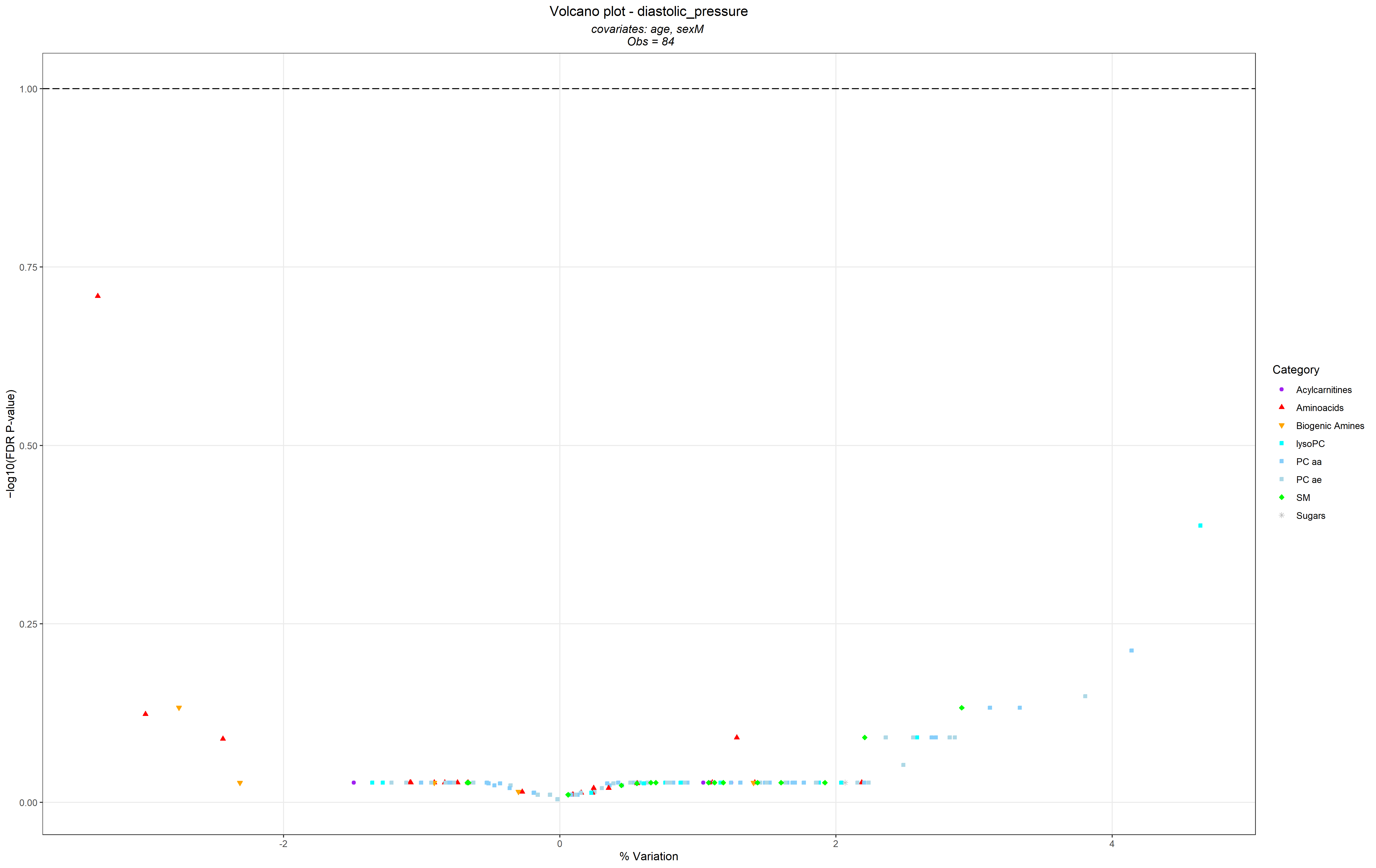

Supplement: Supplementary file 1 [file nutrients-15-00529-s001.zip › 2022-07-12_PRG_Supplementary_material_GF/S11_PRG_lmer_diastolic_pressure.png]

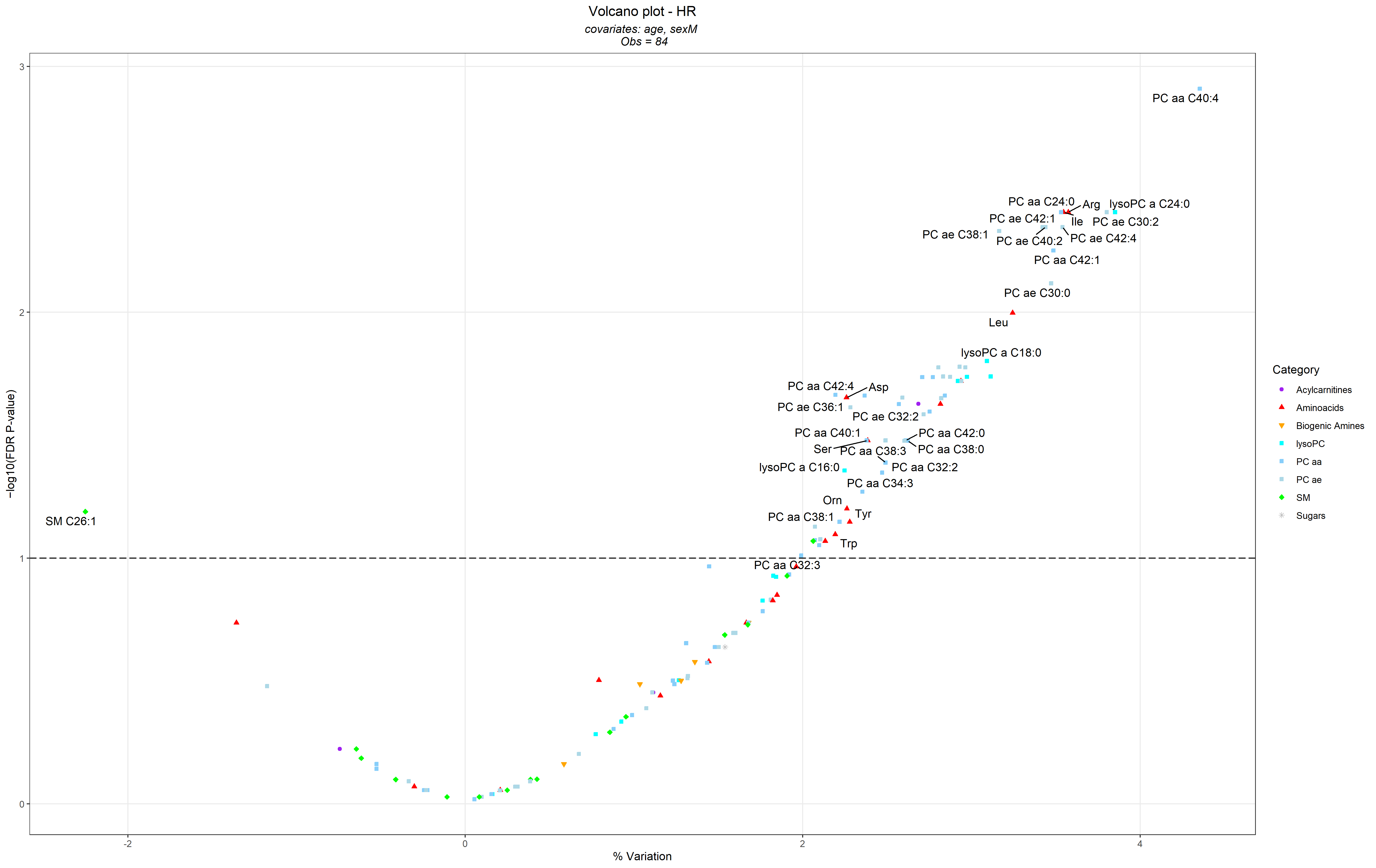

Supplement: Supplementary file 1 [file nutrients-15-00529-s001.zip › 2022-07-12_PRG_Supplementary_material_GF/S12_PRG_lmer_HR.png]

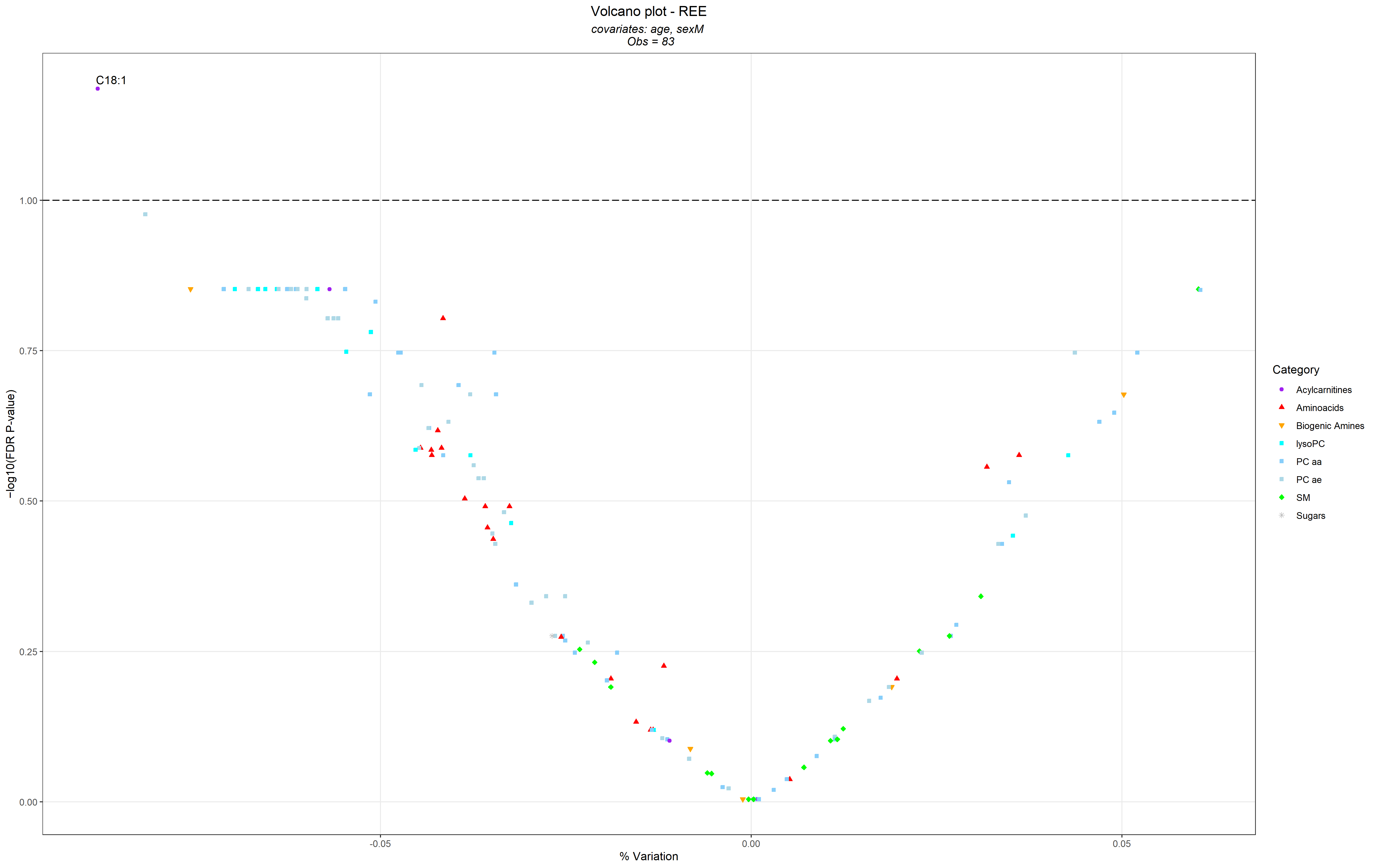

Supplement: Supplementary file 1 [file nutrients-15-00529-s001.zip › 2022-07-12_PRG_Supplementary_material_GF/S13_PRG_lmer_REE.png]

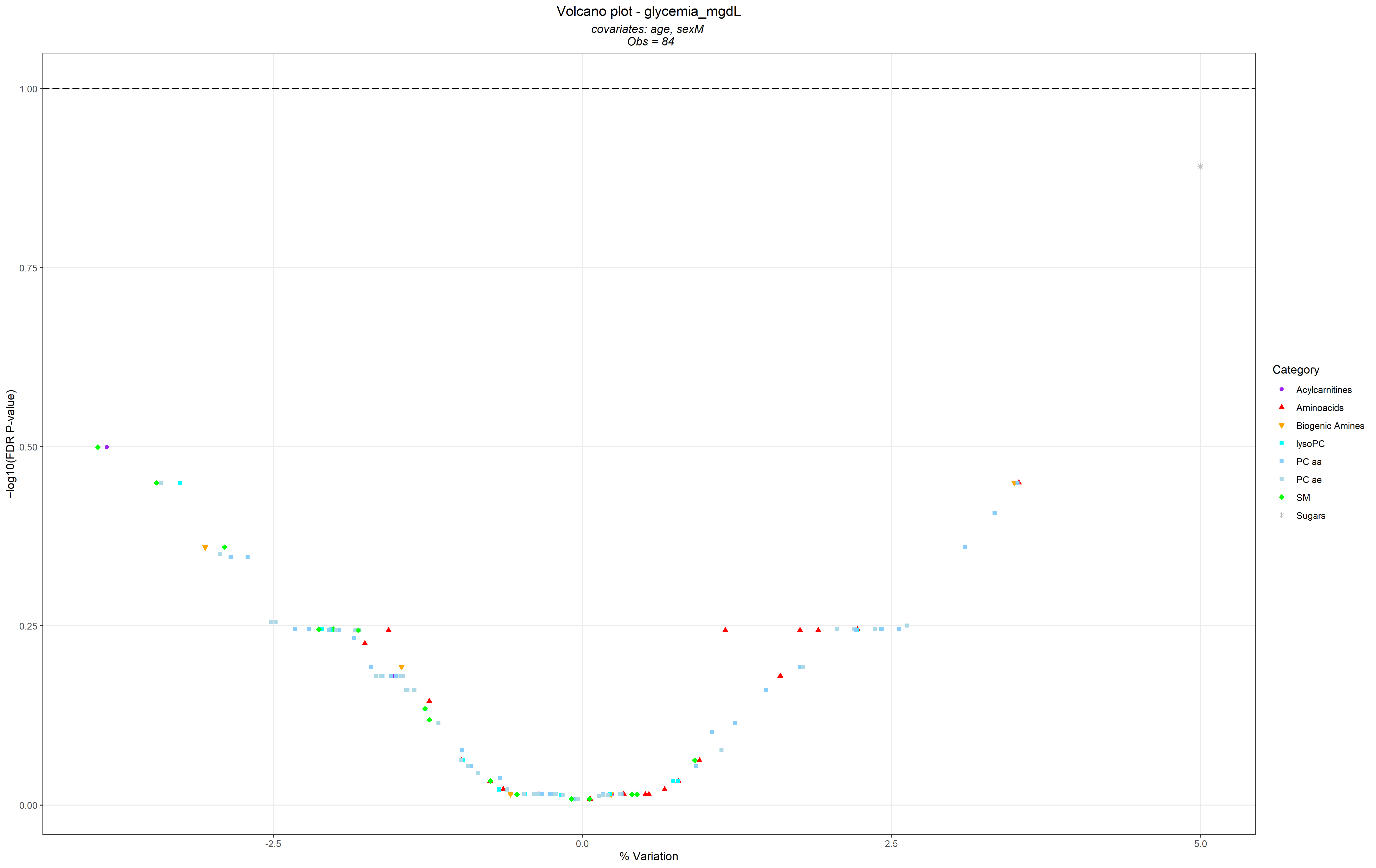

Supplement: Supplementary file 1 [file nutrients-15-00529-s001.zip › 2022-07-12_PRG_Supplementary_material_GF/S14_PRG_lmer_glycemia_mgdL.png]

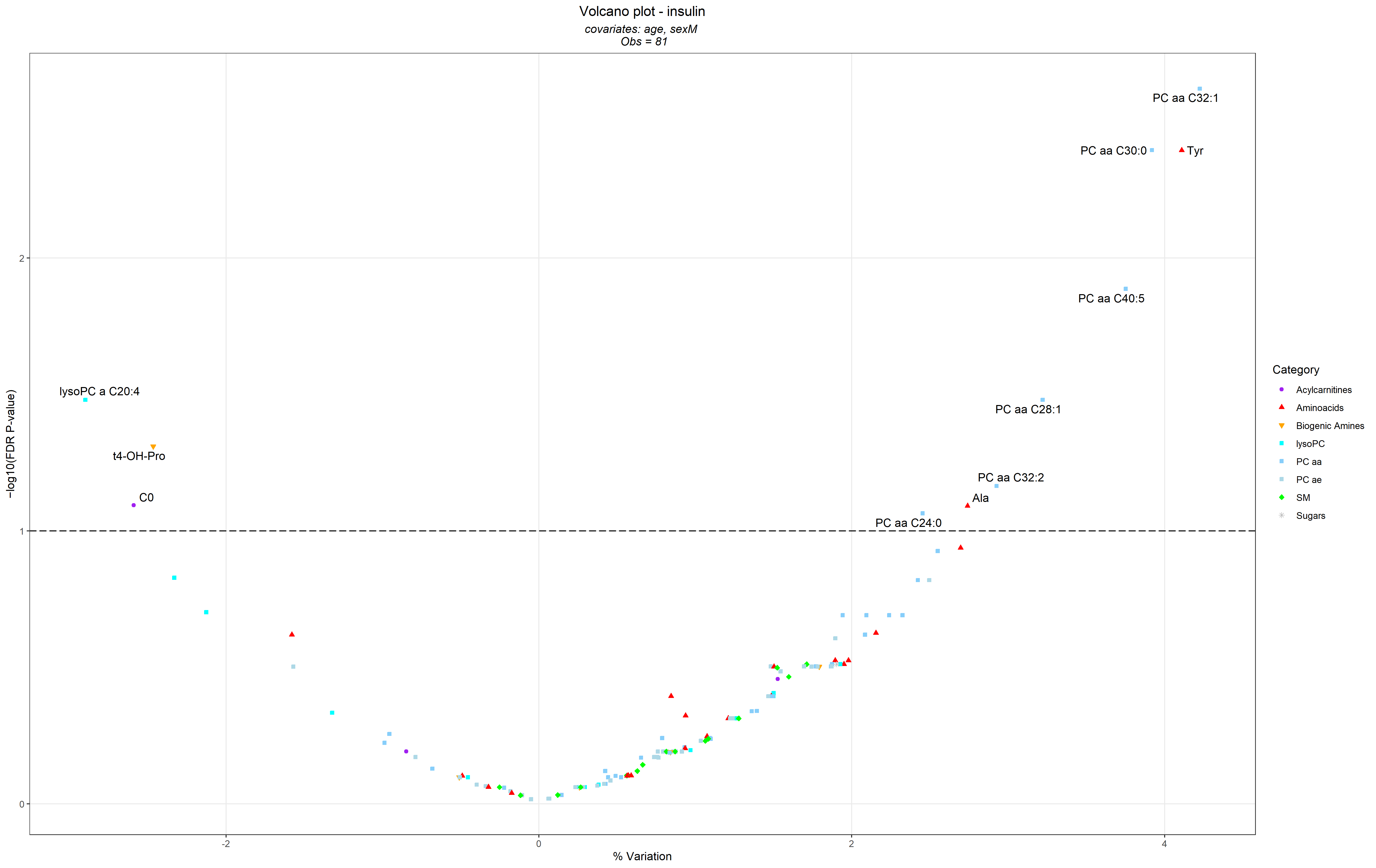

Supplement: Supplementary file 1 [file nutrients-15-00529-s001.zip › 2022-07-12_PRG_Supplementary_material_GF/S15_PRG_lmer_insulin.png]

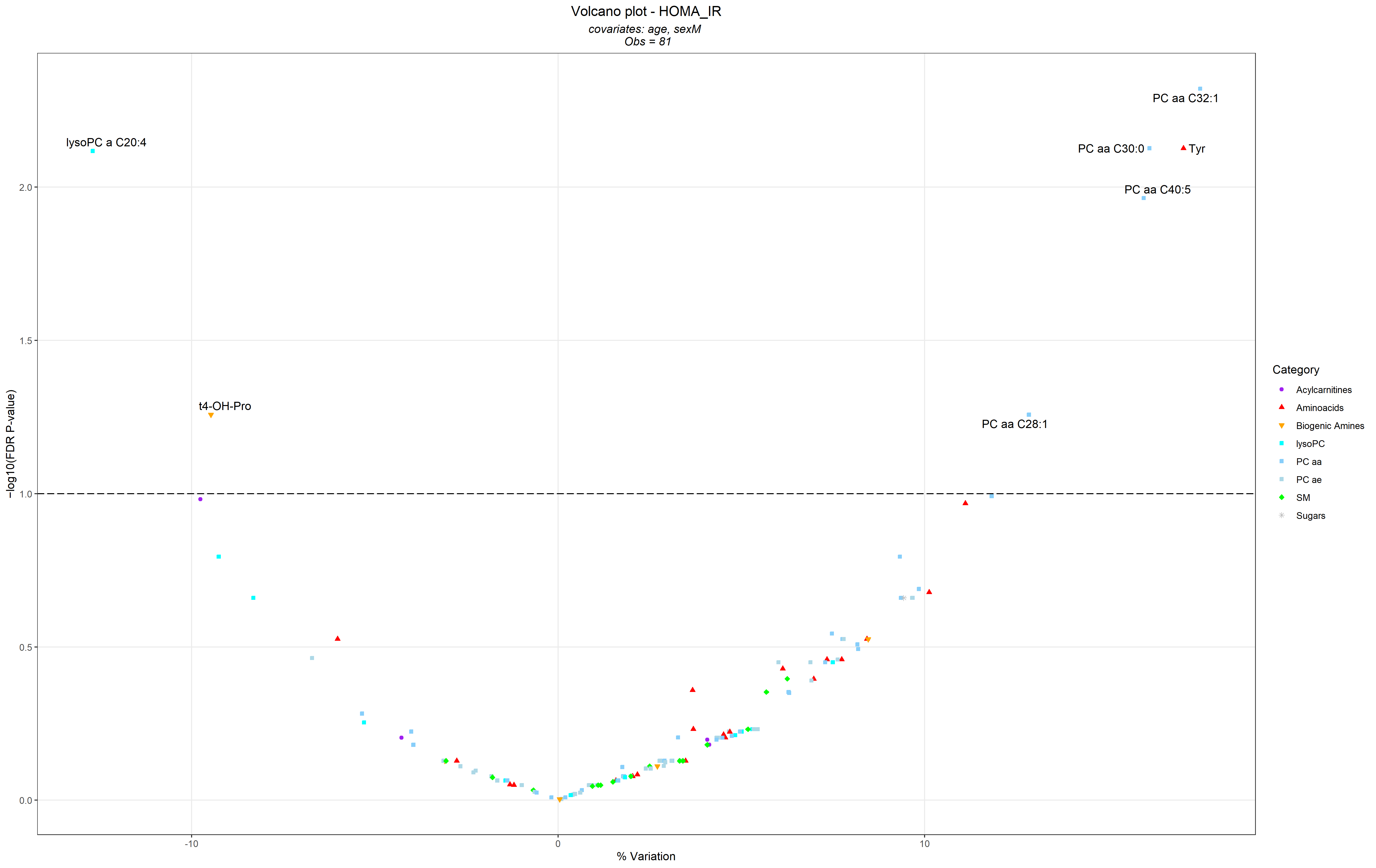

Supplement: Supplementary file 1 [file nutrients-15-00529-s001.zip › 2022-07-12_PRG_Supplementary_material_GF/S16_PRG_lmer_HOMA_IR.png]

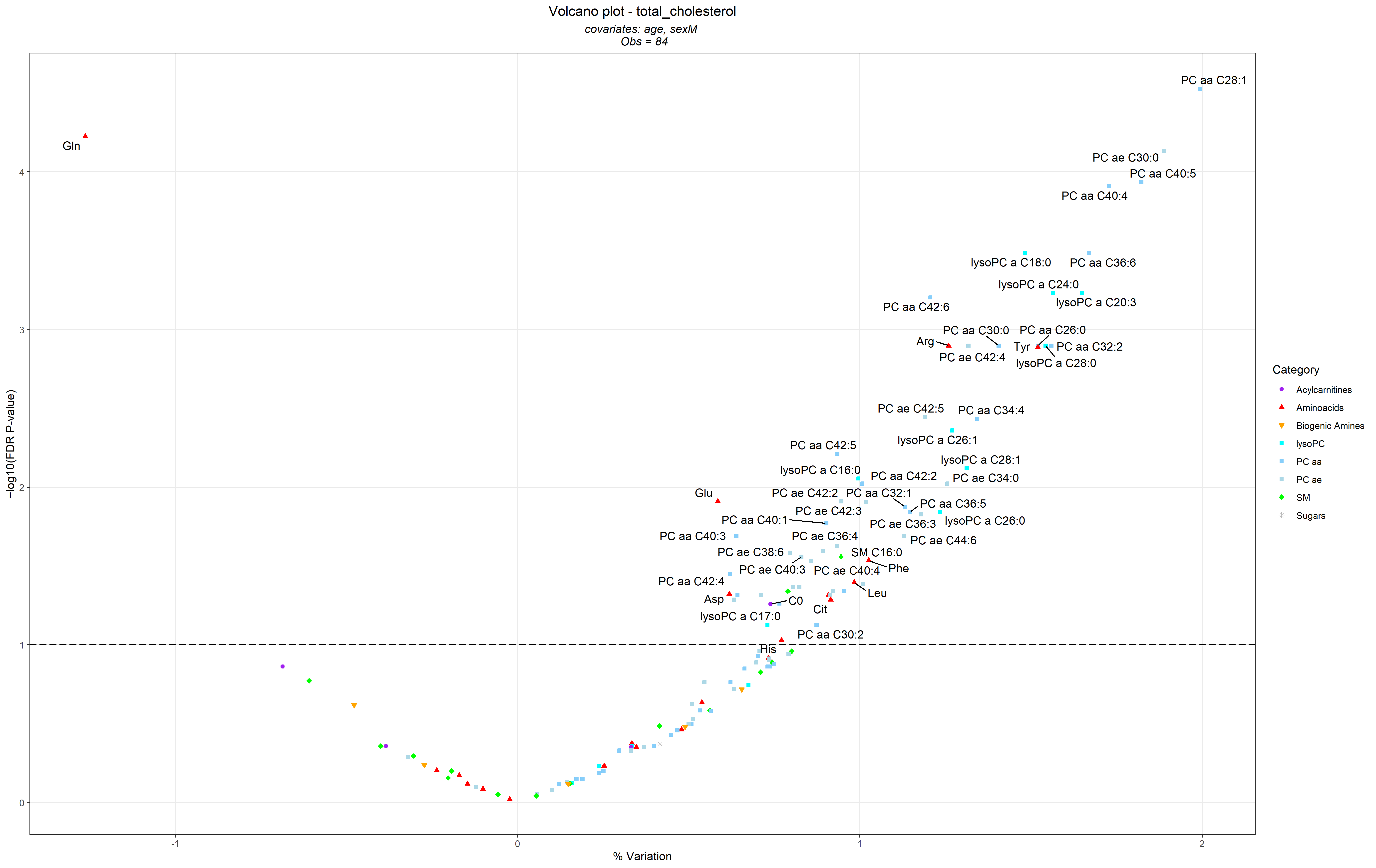

Supplement: Supplementary file 1 [file nutrients-15-00529-s001.zip › 2022-07-12_PRG_Supplementary_material_GF/S17_PRG_lmer_total_cholesterol.png]

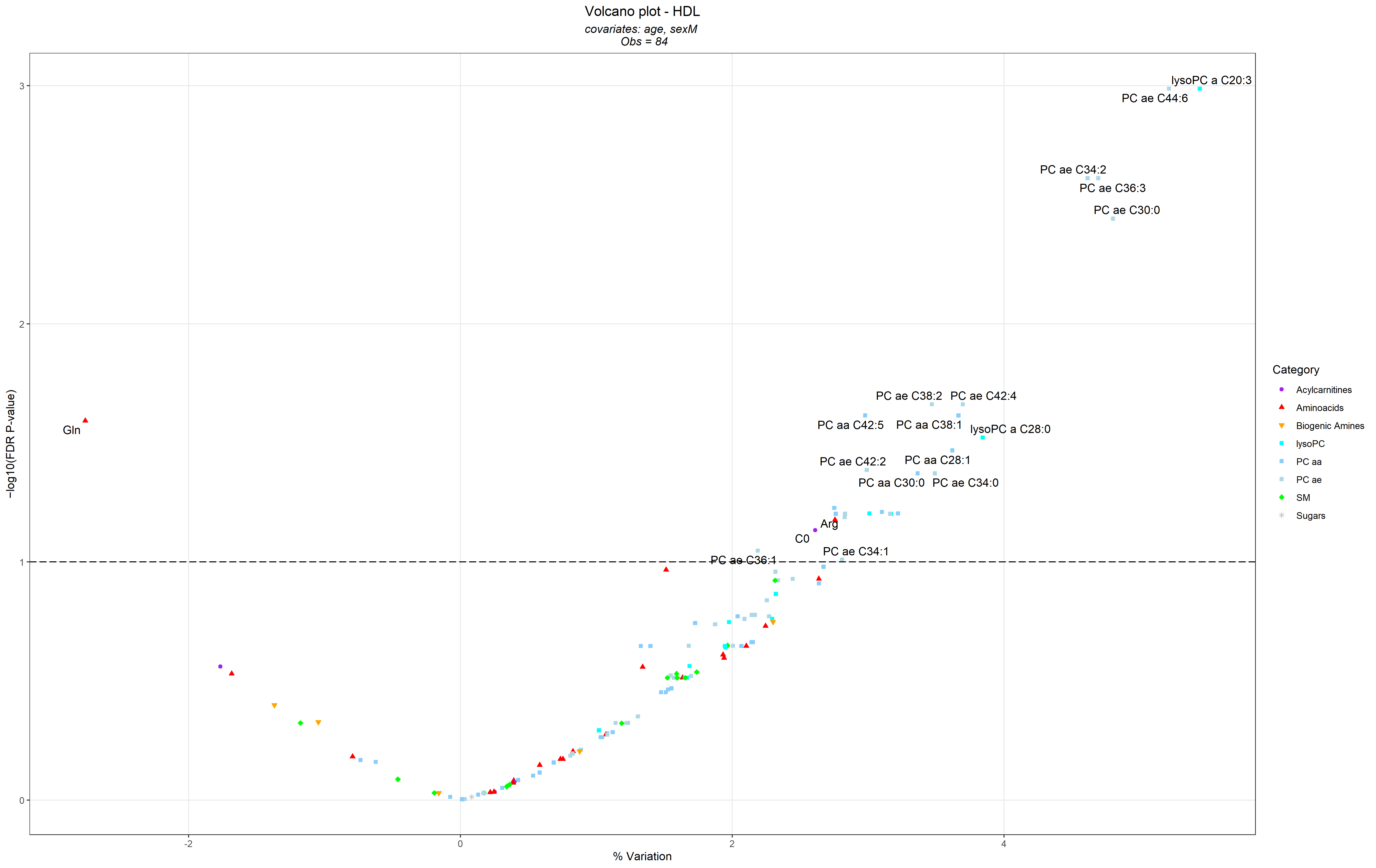

Supplement: Supplementary file 1 [file nutrients-15-00529-s001.zip › 2022-07-12_PRG_Supplementary_material_GF/S18_PRG_lmer_HDL.png]

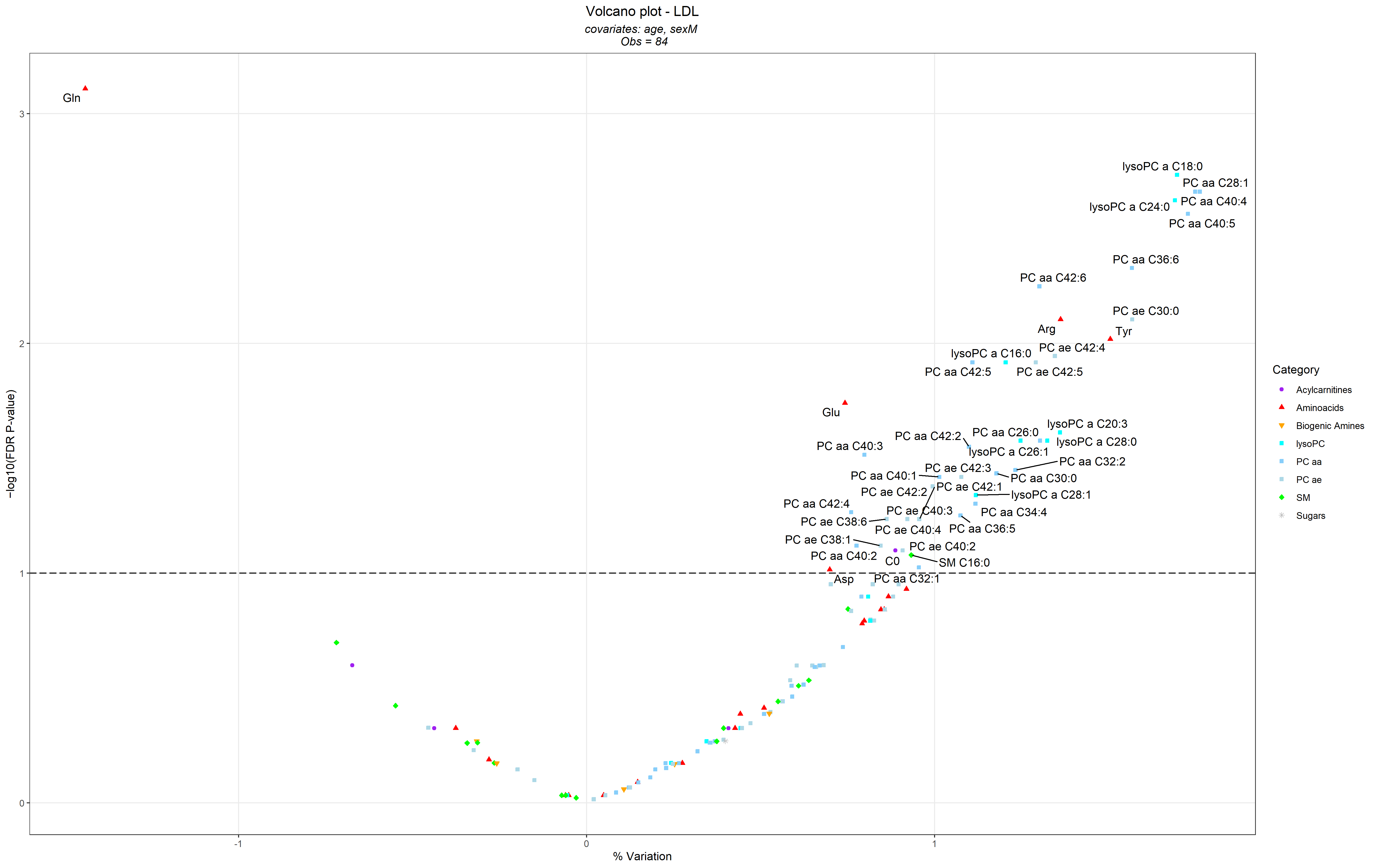

Supplement: Supplementary file 1 [file nutrients-15-00529-s001.zip › 2022-07-12_PRG_Supplementary_material_GF/S19_PRG_lmer_LDL.png]

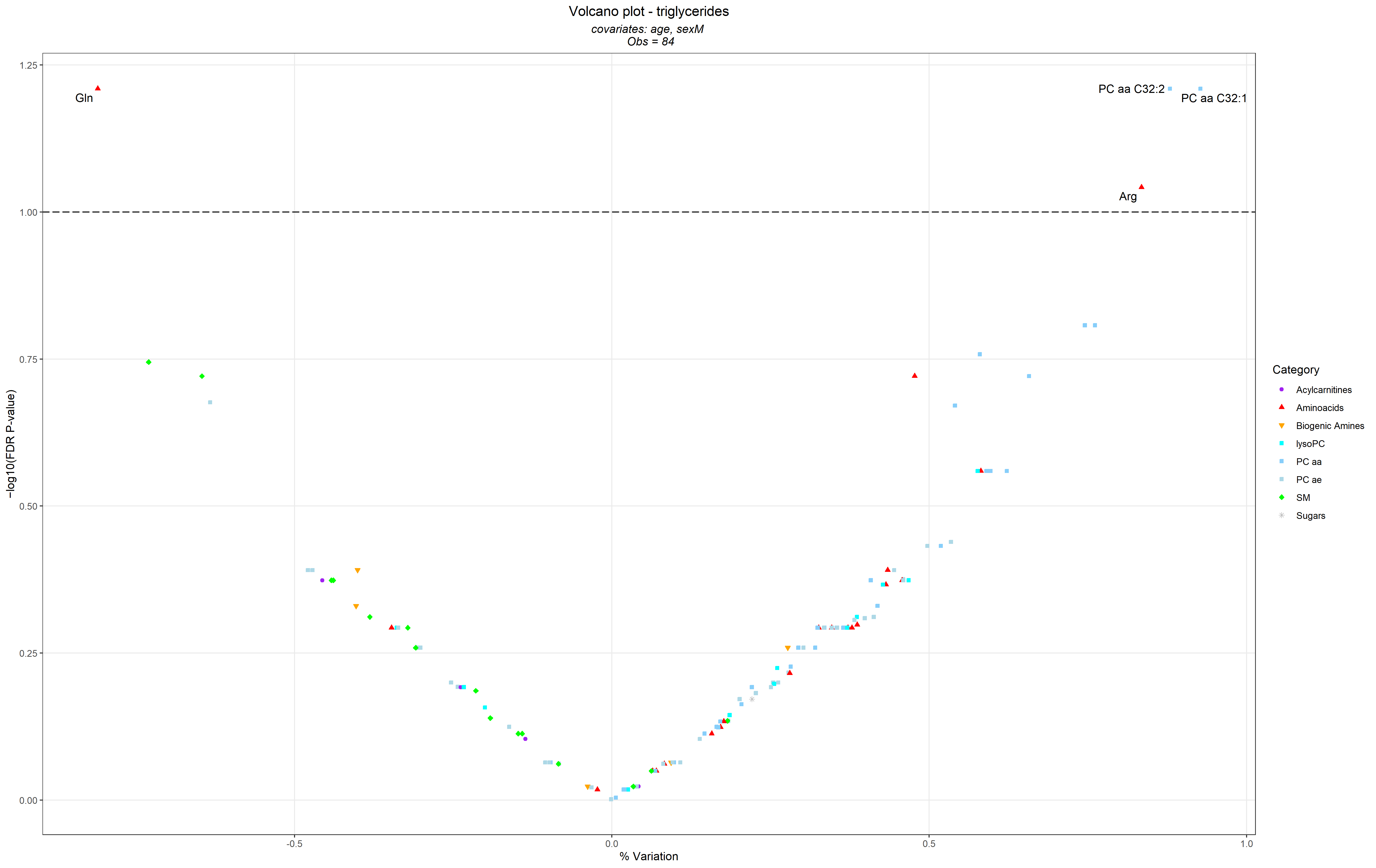

Supplement: Supplementary file 1 [file nutrients-15-00529-s001.zip › 2022-07-12_PRG_Supplementary_material_GF/S20_PRG_lmer_triglycerides.png]

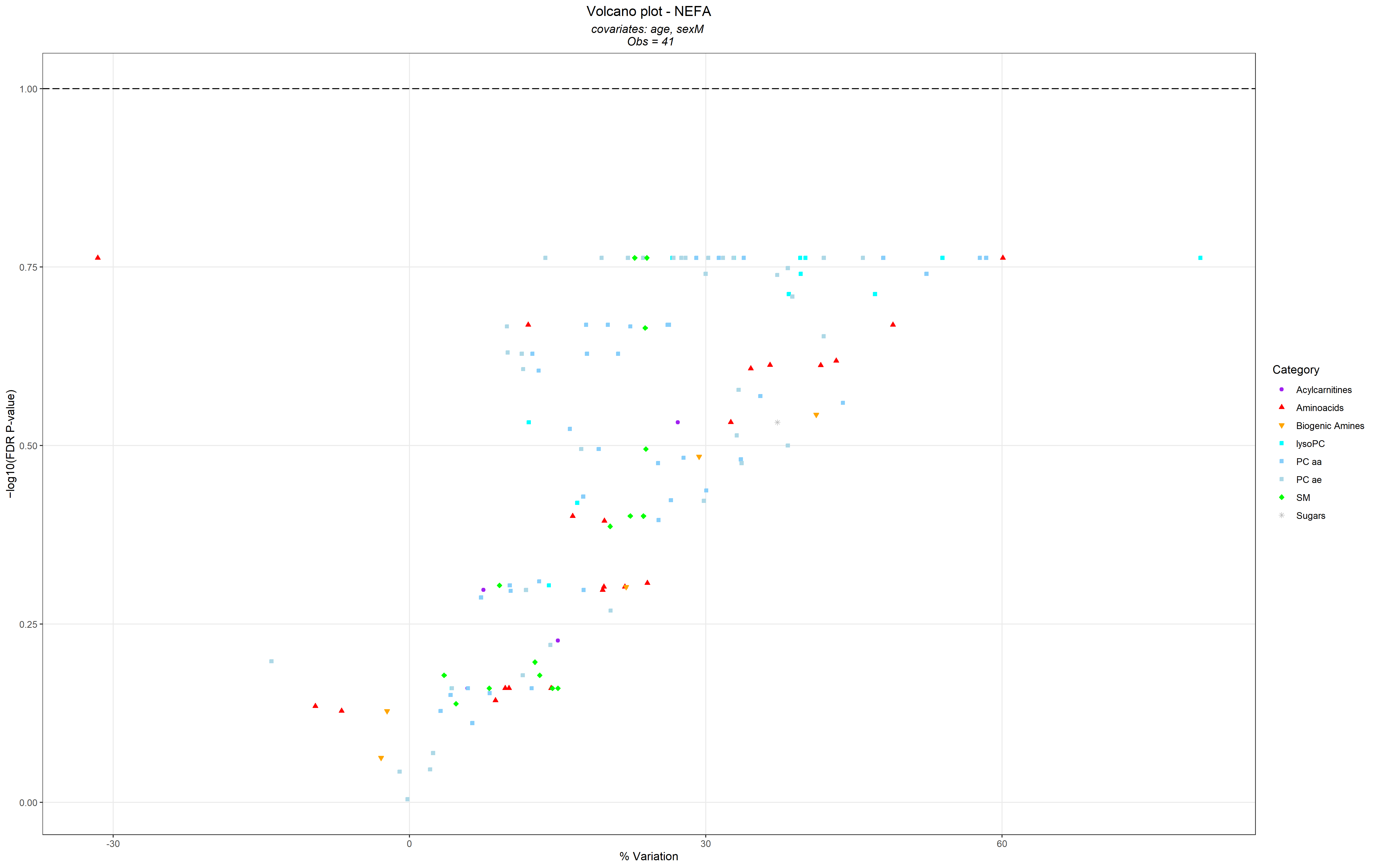

Supplement: Supplementary file 1 [file nutrients-15-00529-s001.zip › 2022-07-12_PRG_Supplementary_material_GF/S21_PRG_lmer_NEFA.png]

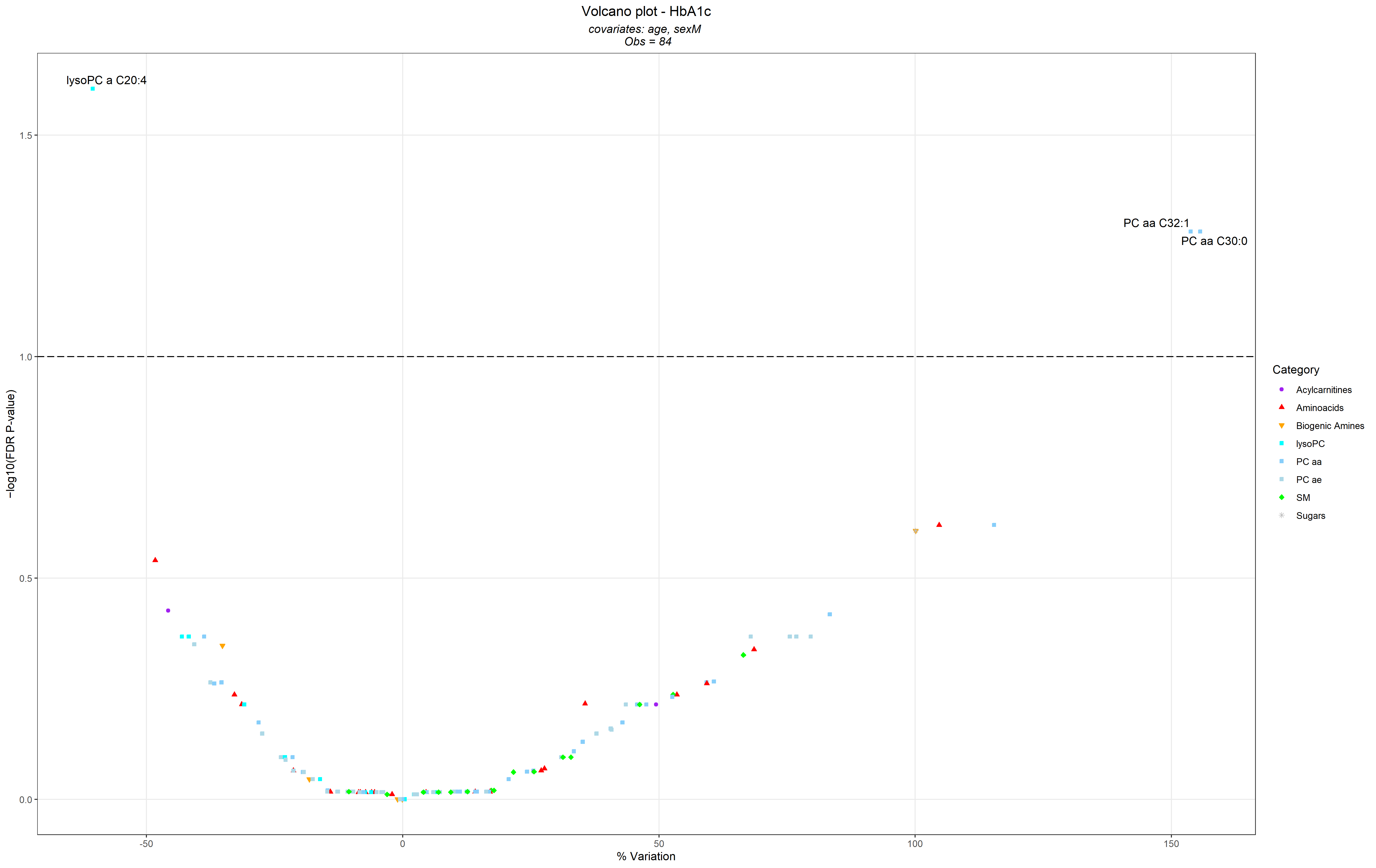

Supplement: Supplementary file 1 [file nutrients-15-00529-s001.zip › 2022-07-12_PRG_Supplementary_material_GF/S22_PRG_lmer_HbA1c.png]

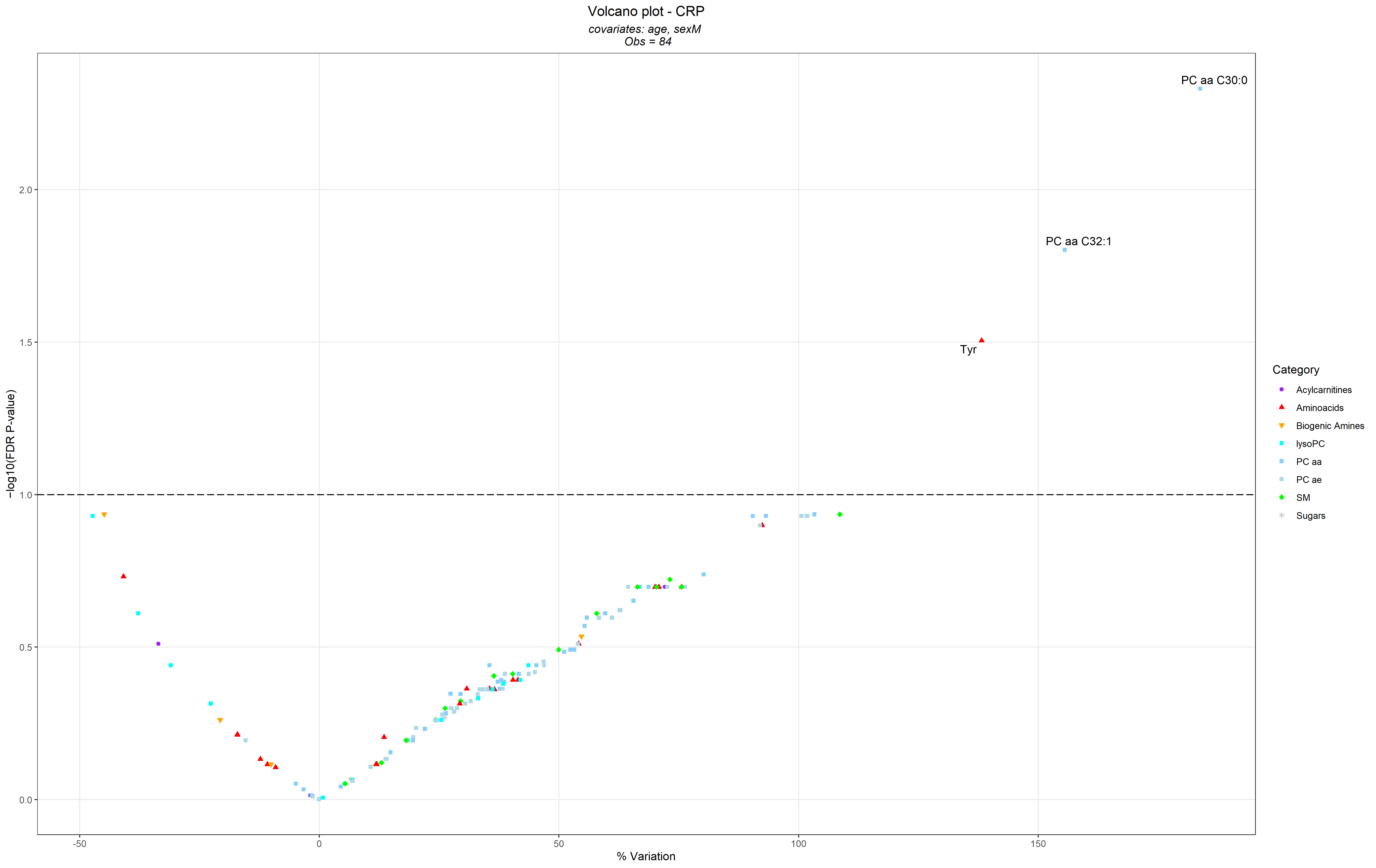

Supplement: Supplementary file 1 [file nutrients-15-00529-s001.zip › 2022-07-12_PRG_Supplementary_material_GF/S23_PRG_lmer_CRP.png]

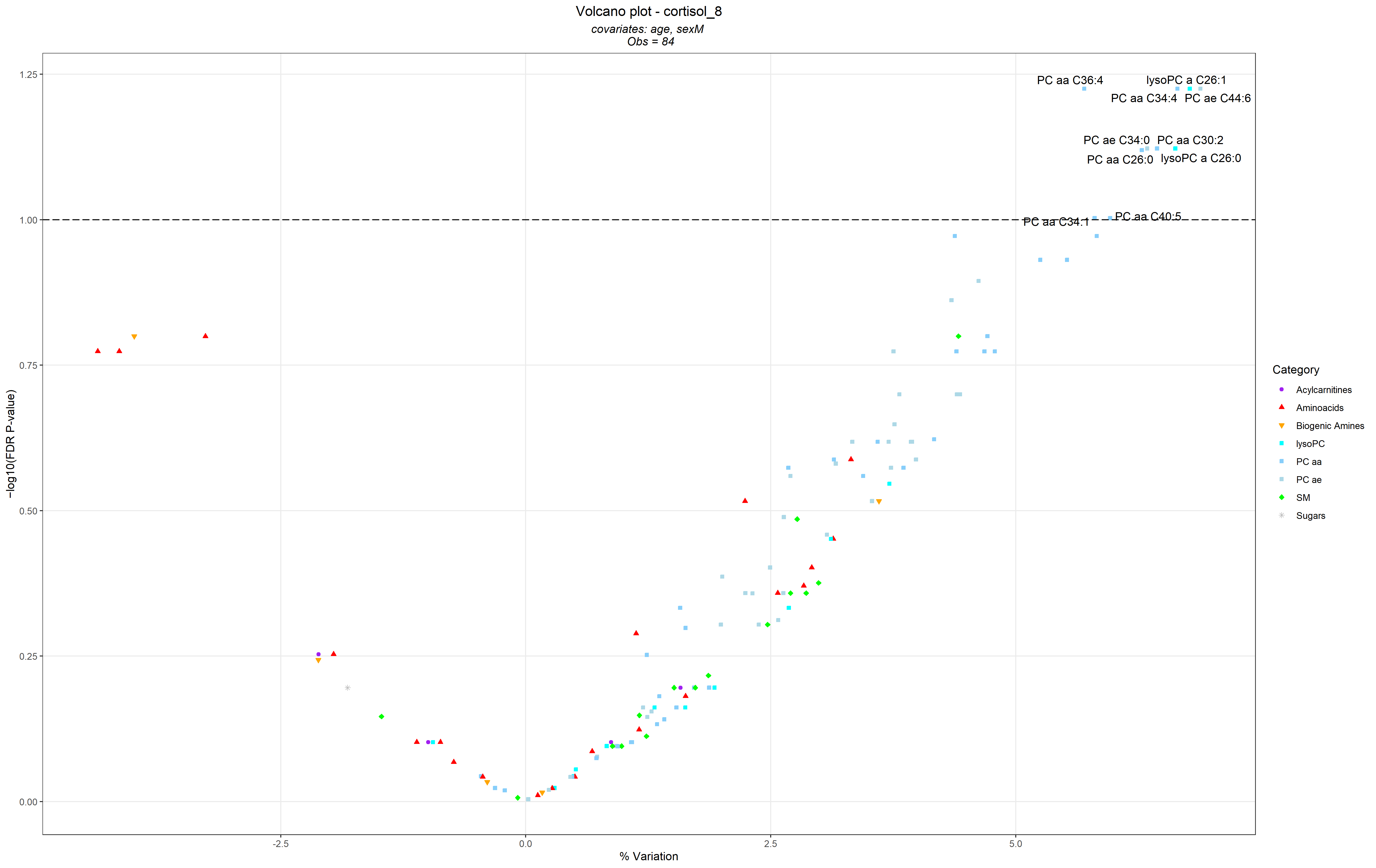

Supplement: Supplementary file 1 [file nutrients-15-00529-s001.zip › 2022-07-12_PRG_Supplementary_material_GF/S24_PRG_lmer_cortisol_8.png]

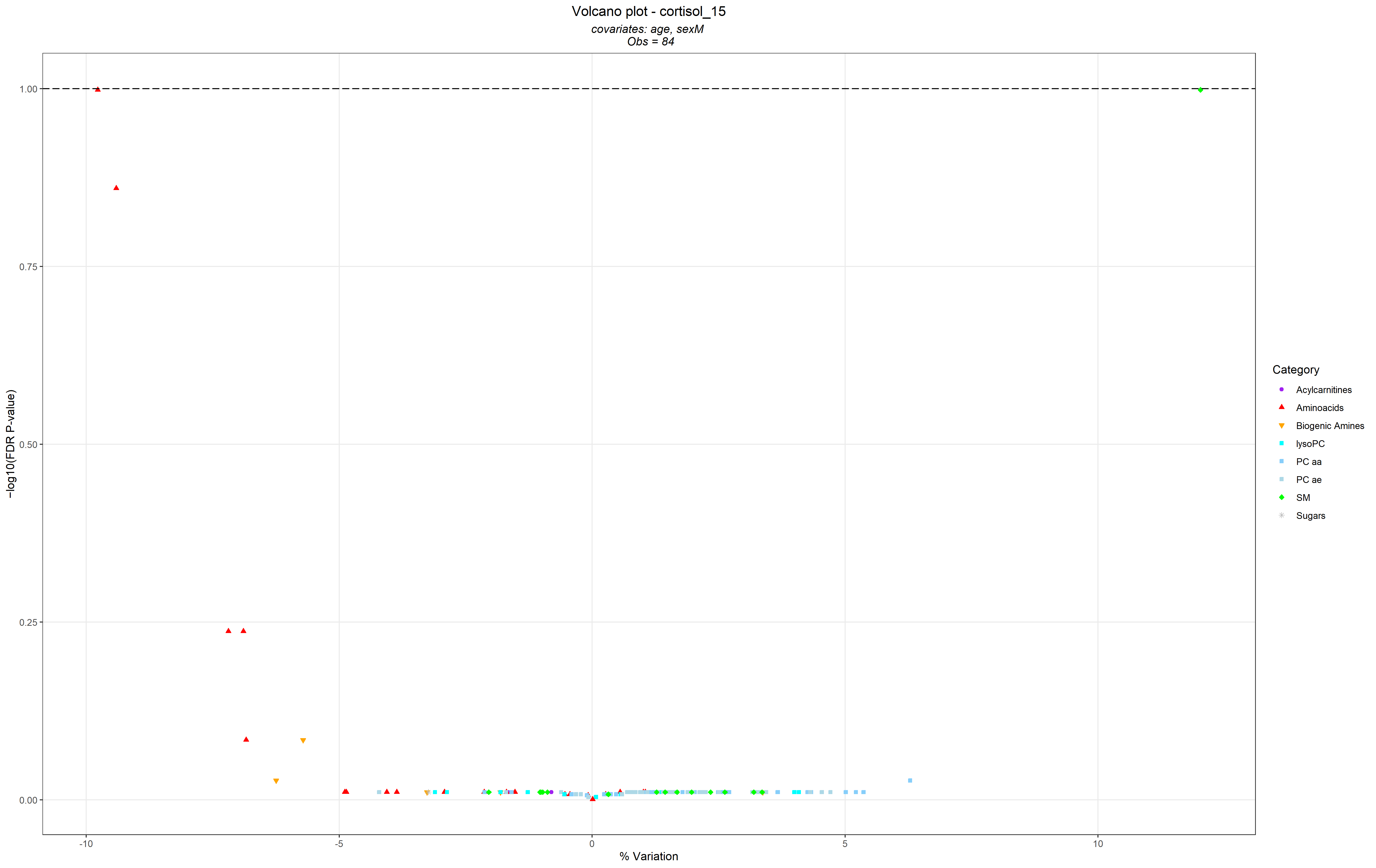

Supplement: Supplementary file 1 [file nutrients-15-00529-s001.zip › 2022-07-12_PRG_Supplementary_material_GF/S25_PRG_lmer_cortisol_15.png]

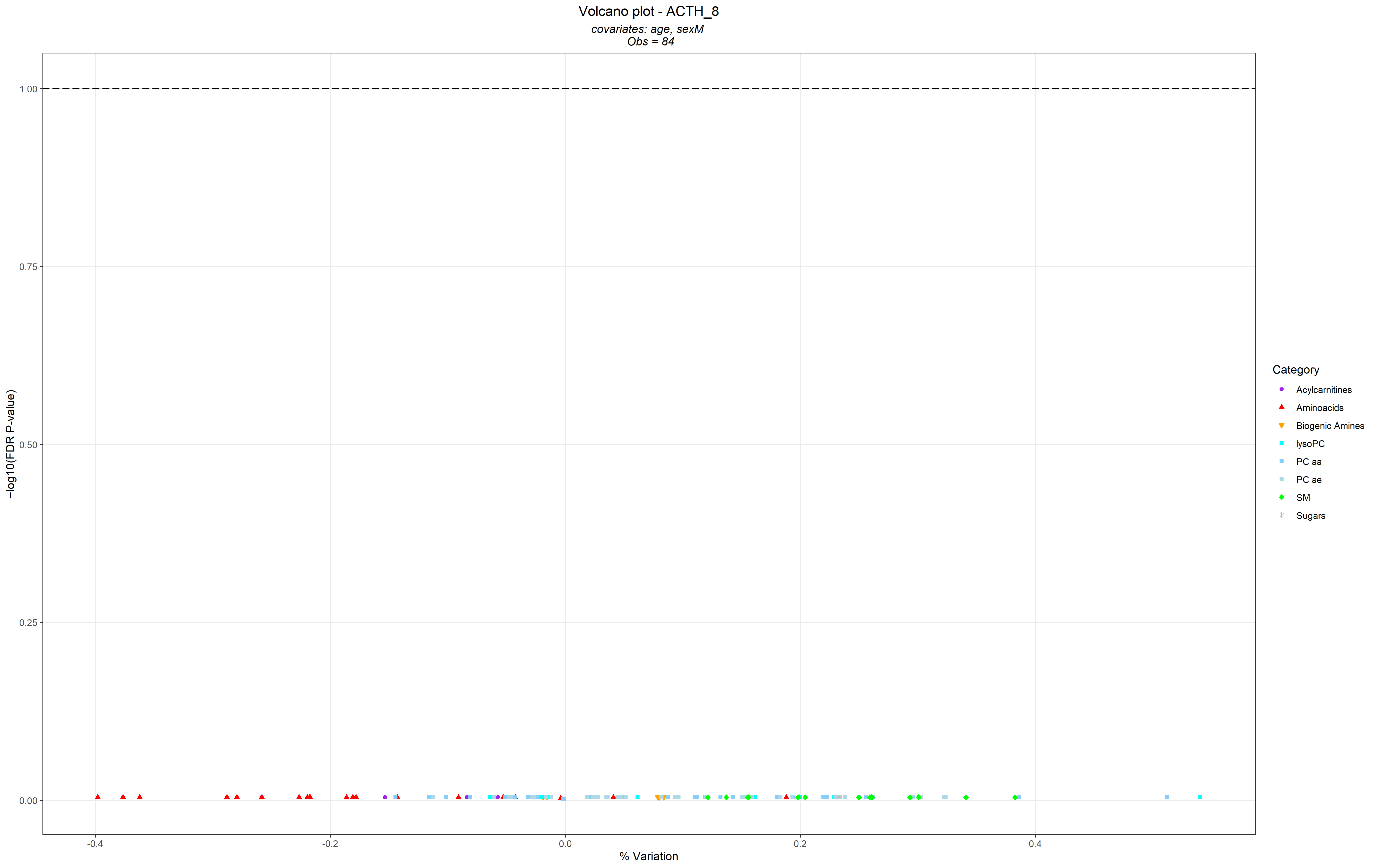

Supplement: Supplementary file 1 [file nutrients-15-00529-s001.zip › 2022-07-12_PRG_Supplementary_material_GF/S26_PRG_lmer_ACTH_8.png]

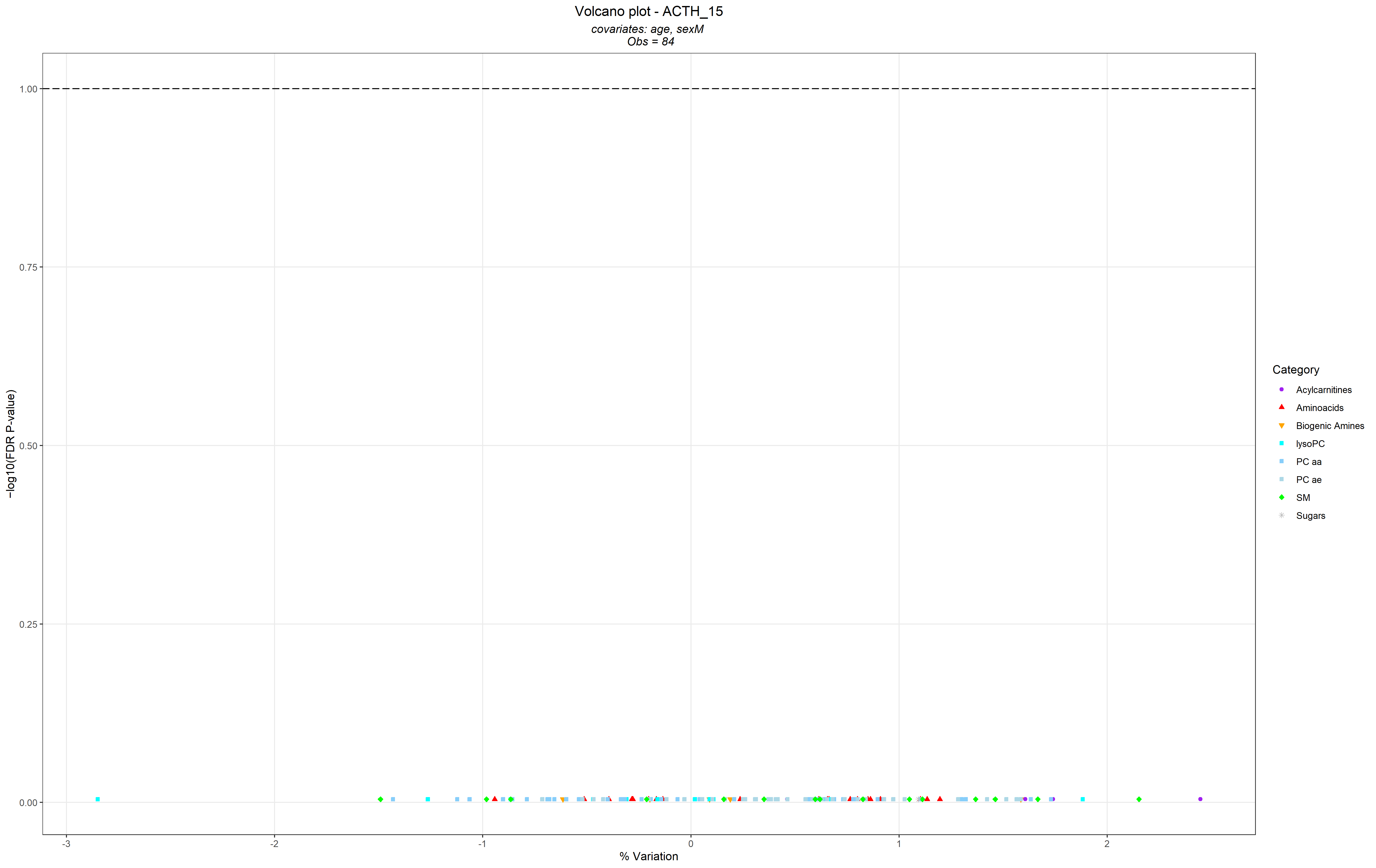

Supplement: Supplementary file 1 [file nutrients-15-00529-s001.zip › 2022-07-12_PRG_Supplementary_material_GF/S27_PRG_lmer_ACTH_15.png]

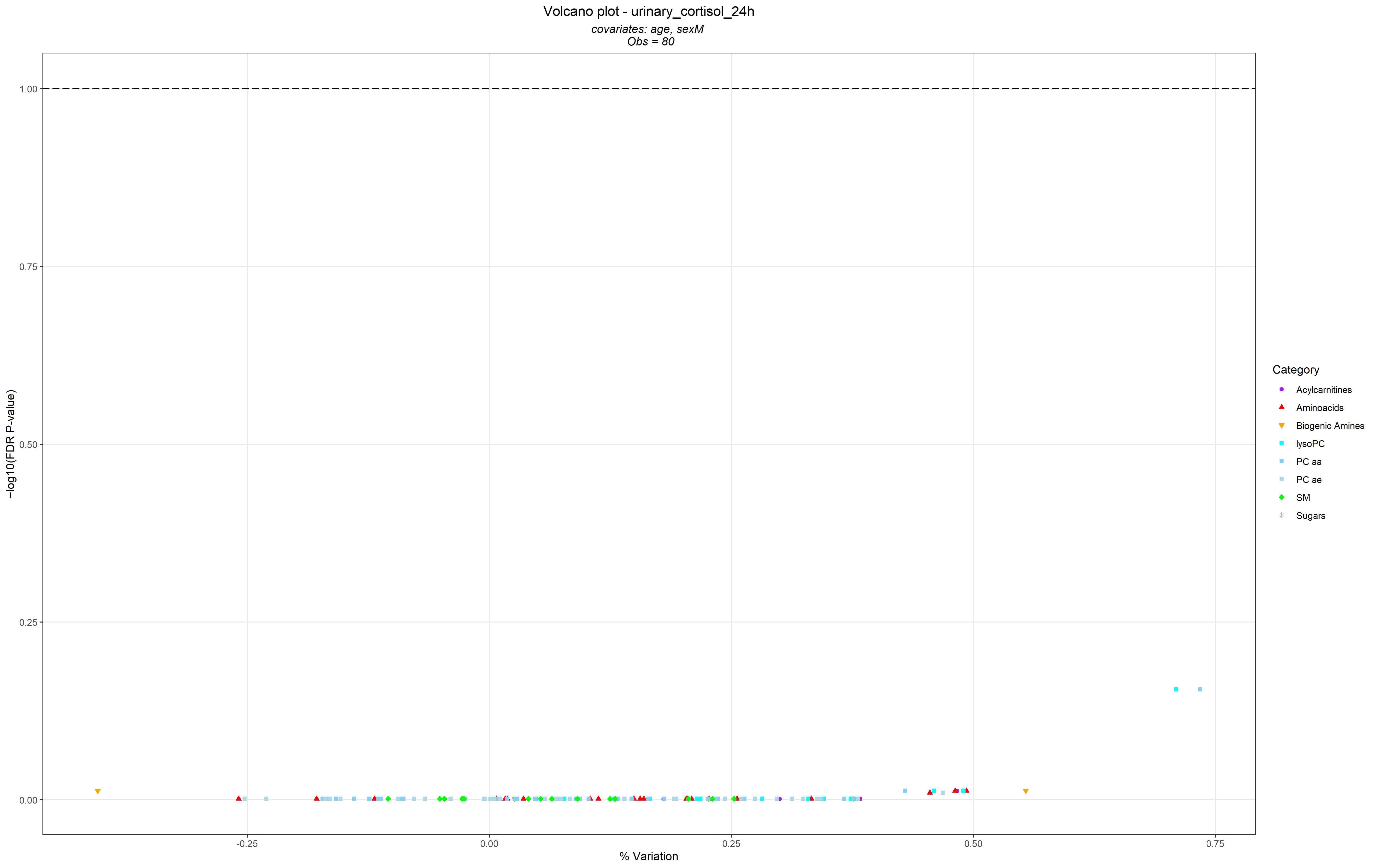

Supplement: Supplementary file 1 [file nutrients-15-00529-s001.zip › 2022-07-12_PRG_Supplementary_material_GF/S28_PRG_lmer_urinary_cortisol_24h.png]

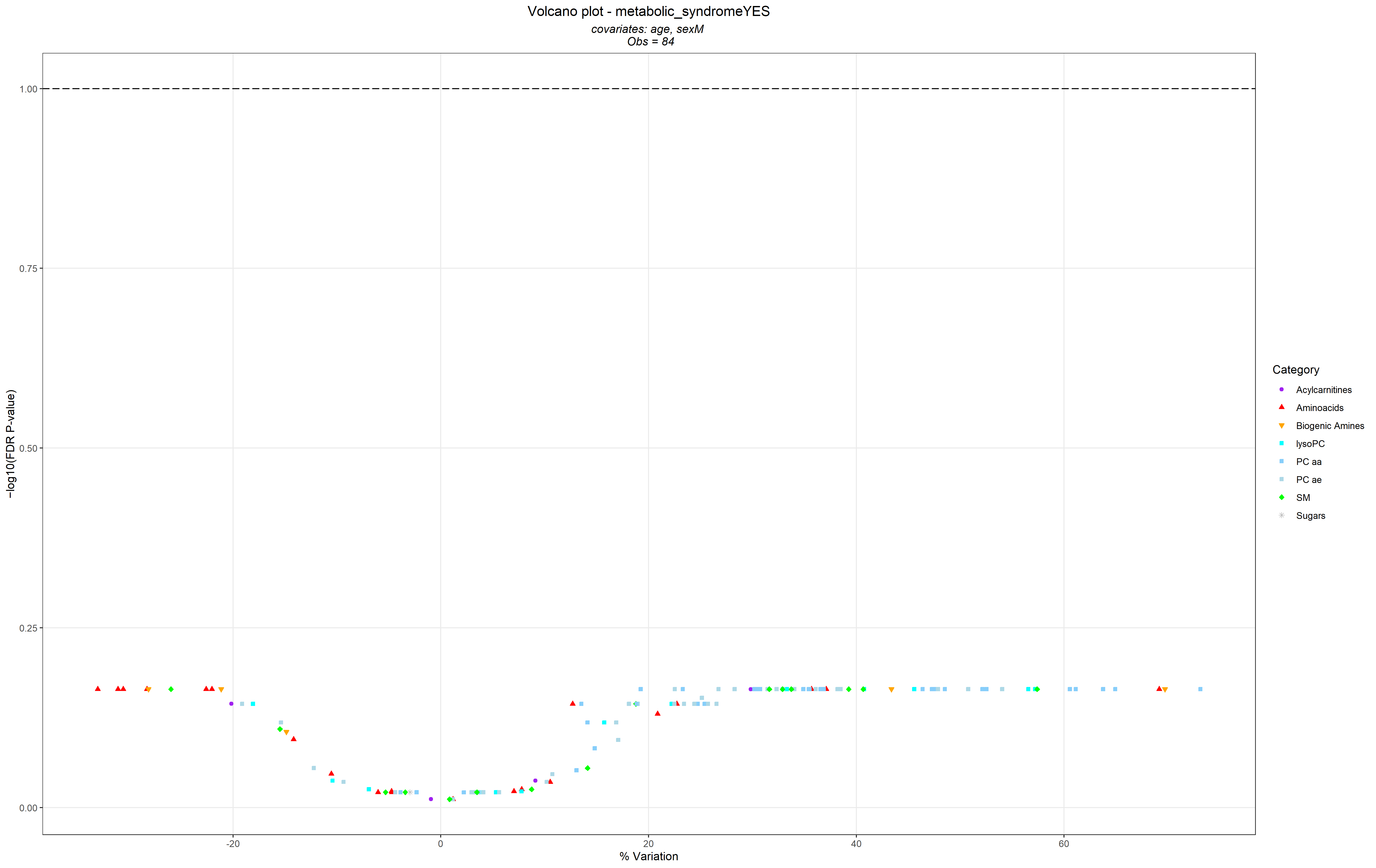

Supplement: Supplementary file 1 [file nutrients-15-00529-s001.zip › 2022-07-12_PRG_Supplementary_material_GF/S29_PRG_lmer_metabolic_syndrome.png]
